# Insertional translocation involving an additional nonchromothriptic chromosome in constitutional chromothripsis: Rule or exception?

**DOI:** 10.1002/mgg3.496

**Published:** 2018-12-18

**Authors:** Nehir Edibe Kurtas, Luciano Xumerle, Ursula Giussani, Alessandra Pansa, Laura Cardarelli, Veronica Bertini, Angelo Valetto, Thomas Liehr, Maria Clara Bonaglia, Edoardo Errichiello, Massimo Delledonne, Orsetta Zuffardi

**Affiliations:** ^1^ Department of Molecular Medicine University of Pavia Pavia Italy; ^2^ Personal Genomics srl, Department of Biotechnologies University of Verona Verona Italy; ^3^ Ospedale Papa Giovanni XXIII Bergamo Italy; ^4^ Laboratorio Analisi CITOTEST. Sarmeola di Rubano Padova Italy; ^5^ Azienda Ospedaliero Universitaria Pisana Pisa Italy; ^6^ Institute of Human Genetics Jena University Hospital Jena Germany; ^7^ Cytogenetics Laboratory, Scientific Institute IRCCS Eugenio Medea Lecco Italy

**Keywords:** chromothripsis, insertional translocations, mosaic, recombinant rearrangements, WGS

## Abstract

**Background:**

Chromothripsis, which is the local massive shattering of one or more chromosomes and their reassembly in a disordered array with frequent loss of some fragments, has been mainly reported in association with abnormal phenotypes. We report three unrelated healthy persons, two of which parenting a child with some degree of intellectual disability, carrying a chromothripsis involving respectively one, two, and three chromosomes, which was detected only after whole‐genome sequencing. Unexpectedly, in all three cases a fragment from one of the chromothripsed chromosomes resulted to be inserted within a nonchromothripsed one.

**Methods:**

Conventional cytogenetic techniques, paired‐end whole‐genome sequencing, polymerase chain reaction, and Sanger sequencing were used to characterize complex rearrangements, copy‐number variations, and breakpoint sequences in all three families.

**Results:**

In two families, one parent was carrier of a balanced chromothripsis causing in the index case a deletion and a noncontiguous duplication at 3q in case 1, and a t(6;14) translocation associated with interstitial 14q deletion in case 2. In the third family, an unbalanced chromothripsis involving chromosomes 6, 7, and 15 was inherited to the proband by the mosaic parent. In all three parents, the chromothripsis was concurrent with an insertional translocation of a portion of one of the chromothriptic chromosomes within a further chromosome that was not involved in the chromothripsis event.

**Conclusion:**

Our findings show that (a) both simple and complex unbalanced rearrangements may result by the recombination of a cryptic parental balanced chromothripsis and that (b) insertional translocations are the spy of more complex rearrangements and not simply a three‐breakpoint event.

## 1 INTRODUCTION

The term chromothripsis was coined to describe a new “all at once” process, identified by genome sequencing techniques as a new driver of tumorigenesis by which, challenging the well‐known mechanism of gradual accumulation of mutations favouring cell duplication/survival, a single catastrophic event of massive shattering and disordered reassembly of one or few chromosomes induced oncogenic lesions (Stephens et al., 2011). Remarkably, chromothripsis was detected in a wide range of cancer entities and recognized to be the hallmarks of those with a poor prognosis (Notta et al., 2016; Rode, Maass, Willmund, Lichter, & Ernst, 2016). Thereafter its discovery in cancerous tissues, a very similar pattern was also observed in germline chromosomal rearrangements both at the balanced and at the unbalanced state although, in general, with a minor number of chromosomal breaks than observed in cancers (Bertelsen et al., 2015; Chiang et al., 2012; Kloosterman et al., 2012; Kloosterman, Guryev, et al., 2011; Kloosterman, Hoogstraat, et al., 2011).

Several mechanisms were postulated for the molecular process of local “shattering and stitching.” The most plausible of these is the physical isolation of chromosomes into micronuclei where the trapped chromosome(s) might undergo extensive DNA fragmentation with double‐stranded breakages. This fragmentation is likely to be due to replication asynchrony with respect to the chromatin within the main nucleus, causing the so‐called premature chromosome condensation of the micronucleus material (Hintzsche et al., 2017). The repair of DNA damage occurs in the following cell cycle, after rupture of the micronucleus membrane and reincorporation of the damaged chromatin into the primary nucleus, as elegantly demonstrated by inducing missegregation and micronucleation of the Y chromosome (Ly et al., 2016). The same authors could also confirmed the NHEJ is the major repair pathway for relegation of the fragmented pieces.

Chromothripsis events, as well as previously reported for complex rearrangements, especially occur at the paternal gametogenesis (De Gregori et al., 2007; Grossmann et al., 2010; Kloosterman et al., 2012; Kloosterman, Guryev, et al., 2011; Kloosterman, Hoogstraat, et al., 2011) and result to be de novo in most cases. This is not surprising since, even in the absence of deletions, the new configuration of the reassembled chromosomes has the potential for disruption of topological chromatin domains (TADs) and alteration of gene expression (Middelkamp et al., 2017), thus leading with high likelihood to phenotypic and behavioral alteration that might interfere with an acceptable social and reproductive life.

In the recent years, a few cases had been reported of balanced chromothripsis in healthy or mildly affected persons whose children, more severely affected, were with recombinant genomic imbalances or even with apparently the same rearrangement present in the parent. In some of these cases, the parental karyotype was initially interpreted as a balanced two‐way simple translocation (Bertelsen et al., 2015; de Pagter et al., 2015). In this study, we illustrate two cases in which we demonstrated that the genomic imbalance present in the index case was derived by parental recombination events between the chromothriptic chromosomes and their normal homologs, although the presence of the parental complex rearrangement was far from predictable in both of them. In the third case, the proband showed a three chromosome chromothripsis event almost identical to that present in the mother in which however a cryptic condition of mosaicism for the rearrangement was demonstrated.

Our findings further demonstrate that chromothripsis events are largely under‐recognized and makes it more than ever current the recommendation to apply systemic molecular investigations, now including whole genome sequencing (WGS), also to the parent from whom the imbalance has been inherited. In fact, this procedure is the only one allowing proper interpretation of apparently “simple” cytogenetic rearrangements (Ciccone et al., 2005). Moreover, our data show that genomic consequence of chromothripsis and its repair processes are not only limited to the single or the few chromosomes undergoing the local shattering and stitching, but also extend to unexpected chromosomes wherein insertional translocations of the chromothriptic fragments may occur.

## 2 MATERIALS AND METHODS

### 2.1 Patients’ samples

Informed consents for the genetic analysis and the publication of the results were obtained from all patients’ parents by the clinicians of the genetics centers to which the parents were still addressing.

### 2.2 Paired‐end whole‐genome sequencing and data analysis

Paired‐end libraries were generated from 2.5 μg DNA isolated from peripheral blood leukocytes of all subjects. The sequencing library is prepared using Illumina's TruSeq DNA polymerase chain reaction (PCR)‐free kit (San Diego, CA, USA) by random fragmentation of the DNA sample with Covaris system, followed by 5′ and 3′ adapter ligation. Libraries were sequenced using the Illumina's HiSeq X Ten with 150PE reads. Reads from the fastq files were mapped to the human reference genome GRCh37/hg19 using Isaac Genome Alignment Software (version iSAAC‐03.16.06.06) (Raczy et al., 2013). In order to identify large deletions and duplications in each chromosome, coverage graphs were created by an in‐house script that uses samtools (with “depth” option) to calculate the average coverage on not overlapping 1,000‐bp windows over the whole chromosome length and gnuplot program to produce the figures. Additionally, copy‐number variations (CNVs) were detected by using CNVnator (Abyzov, Urban, Snyder, & Gerstein, 2011). Structural variants were called by using Lumpy (version 0.2.12) (Layer, Chiang, Quinlan, & Hall, 2014) and Manta (version 0.29.6) (Chen et al., 2016). The breakpoints of predicted structural variants were manually checked in Integrative Genomics Viewer (IGV) genome browser (version 2.3.72) (Robinson et al., 2011), and the derivative chromosomes were reconstructed according to the orientations of discordant paired reads detected on each breakpoint. NGS data of each case were presented according to the International System for Human Cytogenomic Nomenclature (ISCN2016; Table 1).

**Table 1:** Structural Variations detected by NGS

Sample ID	Fragment		Genomic location (hg19)	Size	Status	Sequence characteristics at breakpoint junctions	NGS interpretation
Case 1							
1002‐15 (mother)		3b	Chr3:125695544–133464556	~7.7 Mb	Inversion	‐microhomology of 3 bp (AAC) at chr3:125695543::chr3:133464556 ‐microduplication of 2 bp (AA) at chr3:174483795::chr3:143880433 ‐microduplication and microhomology of 6 bp (CCATTT) at chr3:174483799::chr8:32723879 ‐blunt fusions	hg19xg.[chr3:[pter_cen_125695543::125695544_133464556inv::169592489_174480425inv::174483795_174724061inv::143880433_169592488::133464557_143880432::174724062_qter];g.[chr8:32716995_32723878delinschr3:[174480426_174483799]]
		3c	Chr3:133464557–143880432	~10.4 Mb	Transposition		
		3d	Chr3:143880433–169592488	~25.7 Mb	reference position		
		3e	Chr3:169592489–174480425	~4.8 Mb	inverted transposition		
		3f	Chr3:174480426–174483794	~3.3 k	Translocation		
		3g	Chr3:174483795–174724061	~240 k	inverted transposition		
		Chr8	chr8:32716995–32723878	6.8 kb	Deletion		
Case 2							
280‐16 (father)		6a	chr6:[pter_48385249]	~48.3 Mb	Translocation	‐20 bp deletion of chr6: 154434384–154434403 and 2 bp insertion at chr6:154434404::chr6:48394698 ‐13 bp deletion of chr6:91762540–91762552 at chr6:91762539::chr14:86997986 ‐135 bp deletion of chr14:85427521–85427655 at chr14:85427520::chr14:85953778 ‐1 bp insertion and microhomology of 3 bp (TAT) at chr14:85427656::chr6:91762553 ‐blunt fusion at chr6:154434383::chr6:48385249	hg19xg.[chr6:[154434404_qterinv::48394698_cen_89432920::48390565_48390909inv::89432921_91762539]::chr14:86997986_qter;g.[chr14:[pter_cen_85427520::85427656_85953778inv::chr6:[91762553_154434383::pter_48385249inv]]
		6b	chr6:48385250–48390564	~5.3 k	Deletion		
		6x	chr6:48390565–48390909	344 bp	Transposition		
		6c	chr6:48390910–48394697	~3.7 k	Deletion		
		6d	chr6:48394698–89432920	~41 Mb	reference position		
		6e	chr6:89432921–91762539	~2.3 Mb	reference position		
		6f	chr6:91762553–154434383	~62.6 Mb	Translocation		
		6g	chr6:[154434384_qter]	~16 Mb	inverted transposition		
		14h	chr14:[pter_85427655]	~85.4 Mb	reference position		
		14i	chr14:85427656–85953778	~526 k	Inversion		
		14l	chr14:85953779–86997985	~1 Mb	Translocation		
		14m	chr14:[86997986_qter]	~20 Mb	Translocation		
Case 3							
1960‐16 (Mother)		15a	chr15:[pter_40926862]	~40.9 Mb	reference position	‐microhomology of 3 bp (TGC) at chr15:40926862::chr6:84684894 ‐1 bp deletion of chr6:11134989 and microhomology of 3 bp (CCA) at chr6:11134988::chr7:132596053 ‐Microhomology of 3 bp (TGA) at chr6:123371910::chr6:6793991 ‐11 bp deletion of chr15:40926863–40926873 and 100 bp deletion of chr6:84684893–84684794 at chr15:40926874::chr6:84684793 ‐duplication of 6 bp (AGCCCT) at chr6:6793992::chr6:123371911 ‐19 bp deletion of chr7:132591757–132591775 and microhomology of 3 bp (AGG) at chr6:111343917::chr7:132591776 ‐blunt fusion at chr7:132591756::chr6:111349990	hg19xg.[chr15:[40926874_qterinv]::chr6:[6793992_cen_84684793inv::chr6:[123371911_6qter]];g.[chr6:[pter_111343917]::chr7:132591776]];g.[chr7:[pter_cen_132591756]::chr6:[111349990_123371910::pter_6793991inv]];g.[chr15:[pter_cen_40926862::chr6:[84684894_111349988]::chr7:[132596053_qter]]
		15b	chr15:[40926874_qter]	~61 Mb	Translocation		
		6p	chr6:[pter_6793991]	~6.7 Mb	inversion/translocation		
		6b	chr6:111349990–123371910	~12 Mb	Translocation		
		6x	chr6:111343917–111349988	~6 k	Deleted		
		6a	chr6:84684894–111343917	~26.6 Mb	Translocation		
		6c	chr6: 6793992–84684793	~77.8 Mb	Inversion		
		6q	chr6:[123371911_ qter]	~47 Mb	reference position		
		7a	chr7:[pter_132591756]	~132 Mb	reference position		
		7b	chr7:132591776–132596052	~4 k	Translocation		
		7c	chr7:[132596053_qter]	~26 Mb	Translocation		

### 2.3 Polymerase chain reaction and Sanger sequencing

DNA sequence at breakpoint junctions was constructed according to the human reference genome (GRCh37/hg19) using the UCSC Human Genome Browser. Primers (Sigma‐Aldrich, Darmstadt, Germany) for breakpoints of predicted structural variations were designed using primer3 software (Supporting Information Table  S1). The sufficient PCR products for each breakpoint junction were yielded using GoTaq® G2 Flexi DNA polymerase (Promega) with an elongation times of 1 min for 40 cycles. Sanger sequencing reads were examined by Sequence Scanner™ software v2.0.

### 2.4 Genotyping

Genotyping in case 2 was performed on the amniotic DNA and father's blood DNA. PCR amplification of seven different microsatellites located at chromosomes 6 and 14 was performed with fluorescently labeled primers (5‐Fam and Hex; Sigma‐Aldrich, Darmstadt, Germany; Supporting Information Table  S1). PCR products were analyzed on ABI PRISM® 310 Genetic Analyzer, and the sizes of different alleles were examined using Peak Scanner™ software v2.0.

### 2.5 Fluorescence in situ hybridization

Commercially available probes were used to perform fluorescence in situ hybridization (FISH) on metaphase spreads. In case 1, family trio was analyzed using probe targeting FOXL2 (specific for 3q22.3; Agilent, Santa Clara, CA, USA) and a customized oligo probe (Agilent, Santa Clara, CA, USA) targeting SLC7A14 (specific for 3q26.2). In case 2, in order to define the t(6;14) translocation, fetus (256‐16) and father (280‐16) metaphases were analyzed by the following FISH probes: D6S2523 (specific for 6qter) (Kreatech), D14S1419 (specific for 14qter) (Kreatech), SEC63 (6q21)/SMAD6 (15q22.3) (Kreatech), and D6Z1 (specific for cen6) (Aquarius, Cytocell, Tarrytown, NY, USA). Additional FISH was applied on father (280‐16) metaphases by using a customized oligo probe (Agilent, Santa Clara, CA, USA) targeting fragment 14l (see below), at 14q31.3 (chr14:85953778–86997986). For case 3, FISH was performed on proband (1959/16) and mother (1960‐16) metaphases by whole chromosome painting (WCP) of chromosomes 6, 7, and 15 (Cytocell Ltd, Cambridge, UK) and chromosome 15 telomeric probe (Vysis, Abbott Park, Illinois, USA).

### 2.6 Microarray analysis

In all cases, CGH array (180K and 60K in cases 1 and 2, respectively) and SNP‐CGH array (180K in case 3) were performed according to standard manufacturer's protocols (Agilent Technologies, Santa Clara, CA). All nucleotide positions refer to the Human Genome, Feb 2009 Assembly (GRCh37, hg19). The arrays were analyzed using an Agilent scanner and Feature Extraction v.9.1 software (Agilent Technologies). A graphical overview of the results was obtained using CytoGenomics software.

### 2.7 Phenotypic and genomic evaluation of the outcome of the rearrangements

In order to evaluate sequence characteristics and any possible impact of the breakpoints on patient phenotype, we summarized in Supporting Information Table  S3 the genes, TADs, regulatory elements, and repeat elements at the breakpoints. We checked TADs possibly disrupted by the breakpoints of chromothripsis in neurogenic precursor cells (H1‐NPC; Dixon et al., 2012; a neurodevelopmental phenotype was present in the probands of cases 1 and 3) and lymphoblastoid cells (GM12878; Lieberman‐Aiden et al., 2009) by using web‐based 3D Genome Browser (Rao et al., 2014). As in recently published study (Halgren et al., 2018), we also collected the probability of loss‐of‐function intolerance (pLI) according to the Exome Aggregation Consortium (ExAC; Lek et al., 2016), with high pLI scores (pLI ≥ 0.9) indicating genes extremely intolerant to loss of function, and the corresponding haploinsufficiency (HI) scores (Huang, Lee, Marcotte, & Hurles, 2010) (DECIPHER) with percentages between 0% and 10% indicating genes that are more likely to exhibit haploinsufficiency. Genes at disrupted TADs, which are known to be associated with autosomal dominant disease (OMIM), are also indicated. Repeat elements at the breakpoints are denoted by the UCSC RepeatMasker track. Regulatory elements at the breakpoints (DNA and histone modification, chromatin state, transcription factor sites, DNAseI hypersensitivity sites) were denoted by Encode (ENCODE Project Consortium, 2012; Ernst et al., 2011), VISTA enhancer (Visel, Minovitsky, Dubchak, & Pennacchio, 2007), and UCSF Brain DNA Methylation (Maunakea et al., 2010) tracks (UCSC Genome Browser GRCh37/hg19). In Supporting Information Table  S4, the genes (RefSeq and Ensembl genes 92) encompassed in chromothripsis‐mediated CNVs are reported together with haploinsufficiency scores (DECIPHER) and OMIM association.

### 2.8 Accession number

Structural variants and clinical data of each case have been archived in the Database of Genomic Variants (DGVa, https://www.ebi.ac.uk/dgva/data-download) under accession number estd236.

## 3 RESULTS

### 3.1 Case 1

#### 3.1.1 Family history

The proband (1001‐15), born to nonconsanguineous and currently healthy parents, is a 4‐year‐old male child with multiple phenotypic abnormalities and psychomotor delay. The family history was negative for genetic diseases with the exception of the maternal grandmother affected by bilateral keratoconus. Prenatal ultrasound at 20 weeks of gestation revealed ventriculomegaly and coarctation of the aorta; slight intrauterine growth restriction (IUGR) was documented at 32 weeks of gestation. Invasive prenatal investigation was not performed. He was born by spontaneous delivery at 38 weeks of gestation. His birth weight was 2,405 g (50th percentile), length 48 cm (97th percentile), and OFC 31.5 cm (<50th centile) (Villar et al., 2015). Apgar score was 6 at 1 min, 8 at 5 min, and 9 at 20 min. Ventilatory assistance was not needed. He was hospitalized in the neonatal pathology unit for 10 days because of hypotonia, difficulty in feeding, episodes of desaturation following vomit, and coarse face. In the first months, he showed scarce motor–postural organization without other specific neurological signs and general hypotonia. Independent walking was acquired at 22 months of age. At the age of 2.3 years, he showed bitemporal constriction, hypertelorism, large and prominent eyes with megalocornea (diameter 15 mm), right‐sided monocular deficit, recurrent horizontal nystagmus, hypopigmented fundus with bilateral pale papillae, normal bulbar ultrasound and electrophysiological investigation (ERG and PEV), small nose, and full lips. Thinning of the corpus callosum was documented at ultrasound. Moreover, coarctation of the aorta, dorsal‐lumbar hump in sitting position in the absence of vertebral malformations, bilateral flat feet, and bilateral plantar fibrolipomatous hamartoma were present. He had frequent nocturnal awakenings. Speech therapy was initiated because of psychomotor and language delay. During the last visit to the clinical genetics unit at 3 years of age, the patient was using corrective lenses for megalocorneal astigmatism; minimal dorsal kyphosis was still present. He was able to pronounce about 10 words and made onomatopoeic sounds, has a friendly attitude and was able to execute simple orders, indicated what he wanted, said no and yes with the head, and was able to eat alone even with the cutlery. Parents reported that the child avoided crowded environments.

**Figure 1:**
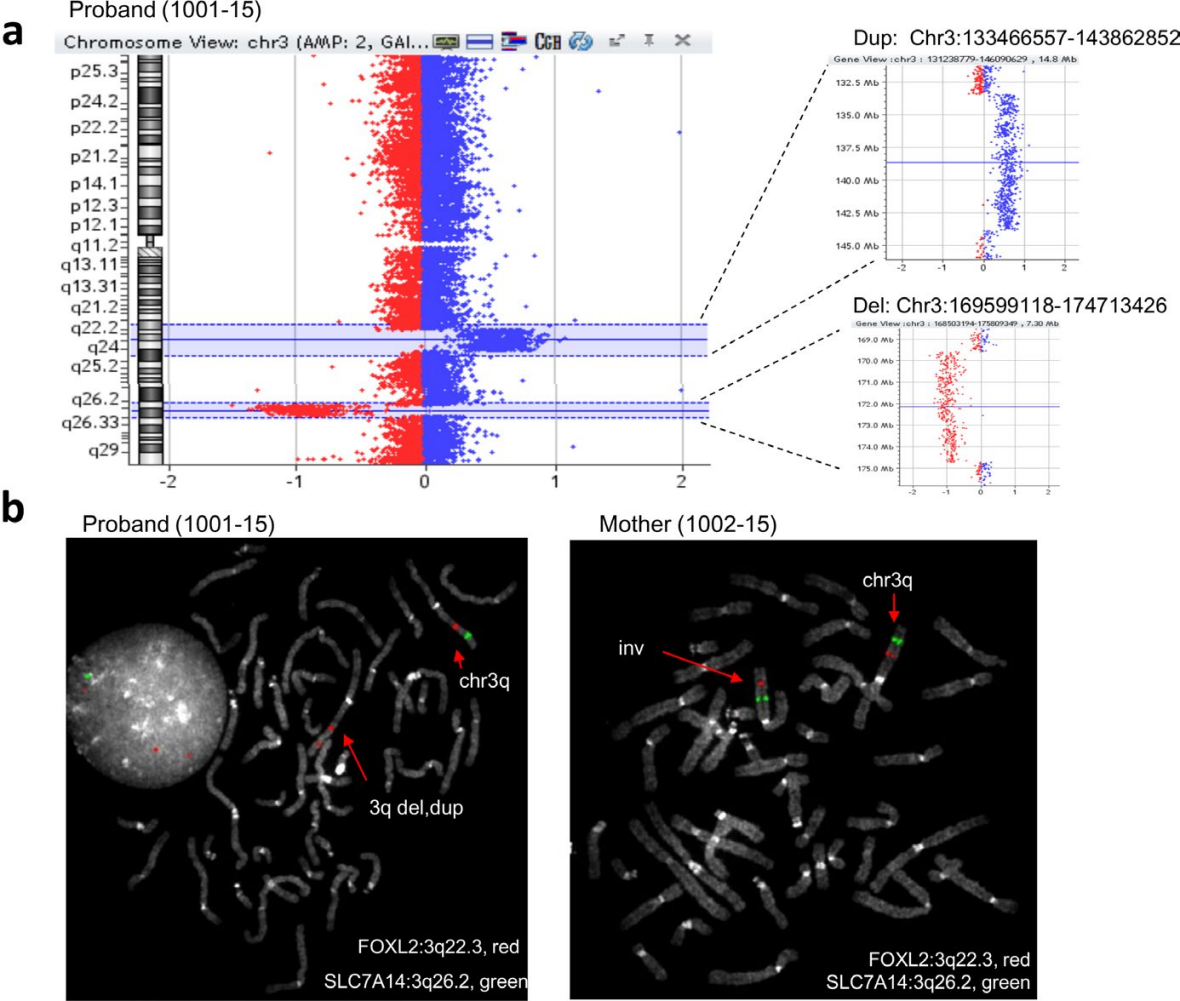
Case 1: Array CGH and FISH findings. (a) Array CGH data displaying de novo 10 Mb duplication and de novo 5 Mb deletion on chromosome 3 of the proband (1001‐15), (b) FISH on metaphases of proband and mother by using probes targeting FOXL2 (3q22.3, red) and SLC7A14 (3q26.2, green) demonstrated the 3q deletion (del) and duplication (dup) in the proband and 3q inversion (inv) in the mother. FISH: fluorescence in situ hybridization

Array CGH data from family trio showed a de novo 10 Mb duplication (Chr3:133466557–143862852) and a de novo 5 Mb deletion (Chr3:169599118–174713426) at the q arm of chromosome 3 of the proband (Figure 1a): 46, XY,arr [GRCh37]3q22.1q24 (133466557_143862852)x3dn, 3q26.2q26.31(169599118_174713426)x1dn. To better understand and confirm these findings, FISH was performed in trio metaphases by using probes targeting FOXL2 (3q22.3, red) and SLC7A14 (3q26.2, green). As a result, the proband showed a karyotype: 46,XY.ish dup(3)(q22.3)(FOXL2++),del3(q26.2)(SLC7A14‐), while the mother, whose karyotype appeared normal, carried an inversion at the 3qter (Figure 1b): 46,XX.ish inv(3)(q22.3q26.2)(FOXL2+, SLC7A14+). To obtain a more complete view of the structural variations in the family, we decided to sequence the whole genome of the proband and the mother by using paired‐end WGS.

#### 3.1.2 Whole genome sequencing of the mother revealed a “balanced chromothripsis”

After the alignment, we obtained an average coverage of 31x in the mother's DNA and 45x in the proband's one. The coverage plot analysis on whole chromosome 3 showed a balanced chromosome 3 in the mother DNA, whereas, as previously demonstrated by array CGH and FISH data, a 10 Mb duplication and a 5 Mb deletion were highlighted in proband's DNA (Figure 1, Supporting Information Figure  S1). According to LUMPY and MANTA analysis, the q arm of chromosome 3 of the mother was shattered by seven breakpoints producing eight fragments named as 3a, 3b, 3c, 3d, 3e, 3f, 3g, and 3h. In order to reconstruct the derivative chromosome 3, the orientation of paired reads on each breakpoint was identified (Supporting Information Figure  S2a) and the interpretation confirmed on Sanger sequencing (Supporting Information Figure  S2b): Both the inverted orientation of fragments 3b, 3e, and 3g and the changing in their order implied a “balanced chromothripsis” event at chromosome 3q of the mother (Table 1). Additionally, fragment 3f was predicted and confirmed to be inserted into chromosome 8 where a 6.8 kb deletion (chr8:32716995–32723878) was highlighted by a decreased coverage of the region (Supporting Information Figure  S3). In contrast, in proband's genome, the same 6.8 kb chromosome 8 region was in balanced state (Supporting Information Figure  S3).

#### 3.1.3 Characterization of breakpoints of chromosome 3 by Sanger sequencing

**Figure 2:**
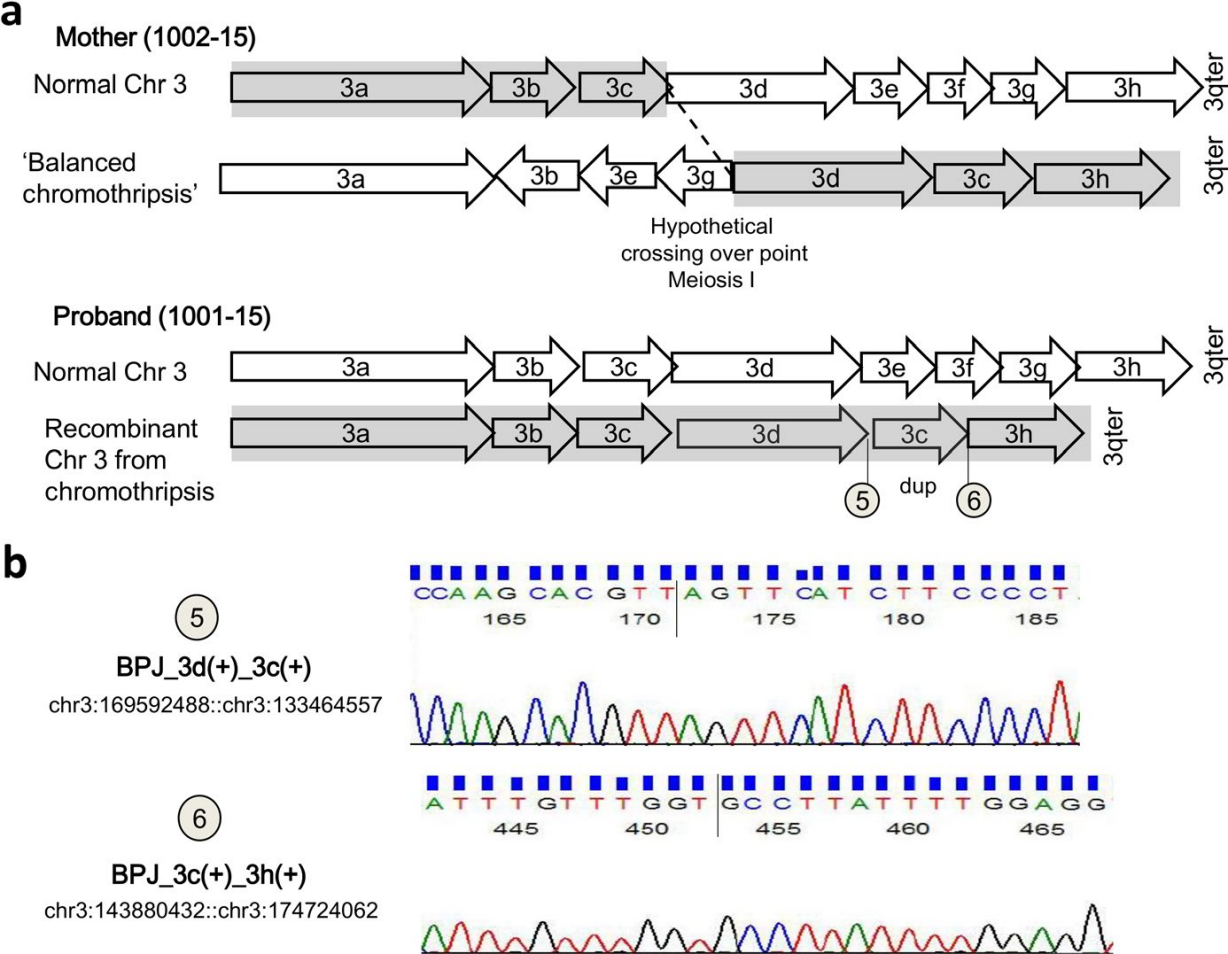
Case 1: schematic view of the hypothetical recombination event during maternal meiosis (fragment size not in scale). (a) Recombination in maternal meiosis I occurred between normal and derivative chromosome 3 at a hypothetical crossover point, producing a recombinant chromosome 3 with the duplication (dup) of fragment 3c (consistent with the duplicated chromosomal location Chr3:133466557–143862852 of the proband, as detected by array CGH analysis) and deletion of fragments 3e‐3f‐3g (consistent with the deleted chromosomal location Chr3:169599118–174713426 of the proband, as detected by array CGH analysis). (b) Sanger confirmation of inherited breakpoints junctions, BPJ_3d(+)_3c(+) (chr3:169592488::chr:133464557, number 5) and BPJ_3c(+)_3h(+) (chr3:143880432::chr3:174724062, number 6) on proband (1001‐15) is shown. See also Supporting Information Figure S2 for all the fusion junctions validated on mother's DNA

All breakpoint junctions, namely BPJ_3a(+)_3b(−), BPJ_3b(−)_3e(−), BPJ_3e(−)_3g(−), BPJ_3g(−)_3d(+), BPJ_3d(+)_3c(+), BPJ_3c(+)_3h(+), and the fusion junctions of fragment 3f insertion into chromosome 8, BPJ1_chr8_3f(+) and BPJ2_3f(+)_chr8, were confirmed and fully characterized by Sanger sequencing (Supporting Information Figure  S2b). Out of the eight fusion junctions detected in the mother, the proband (1001–15) inherited the identical BPJ_3d(+)_3c(+) and BPJ_3c(+)_3h(+) fusion junctions, indicating maternal crossover occurrence between the normal and the derivative chromosome 3 (Figure 2a). Additionally, the exact breakpoints of the 3q proband's deletion (fragments 3e, 3f, and 3g, Chr3:169592489–174724061) and duplication (fragment 3c, Chr3:133464557–143880432) were defined (Figure 2b). The breakpoints’ signatures indicated NHEJ mechanisms with blunt fusions (five out of eight BPJs), microduplication (two out of eight), and microhomology (two out of eight; Supporting Information Figure  S2b).

Screening of the grandmaternal parents for the SNPs detected in the mother‐specific fusion junctions revealed the maternal origin of the chromothripsed chromosome 3 of the mother (1002‐15; Supporting Information Figure  S4).

### 3.2 Case 2

#### 3.2.1 Family story

**Figure 3:**
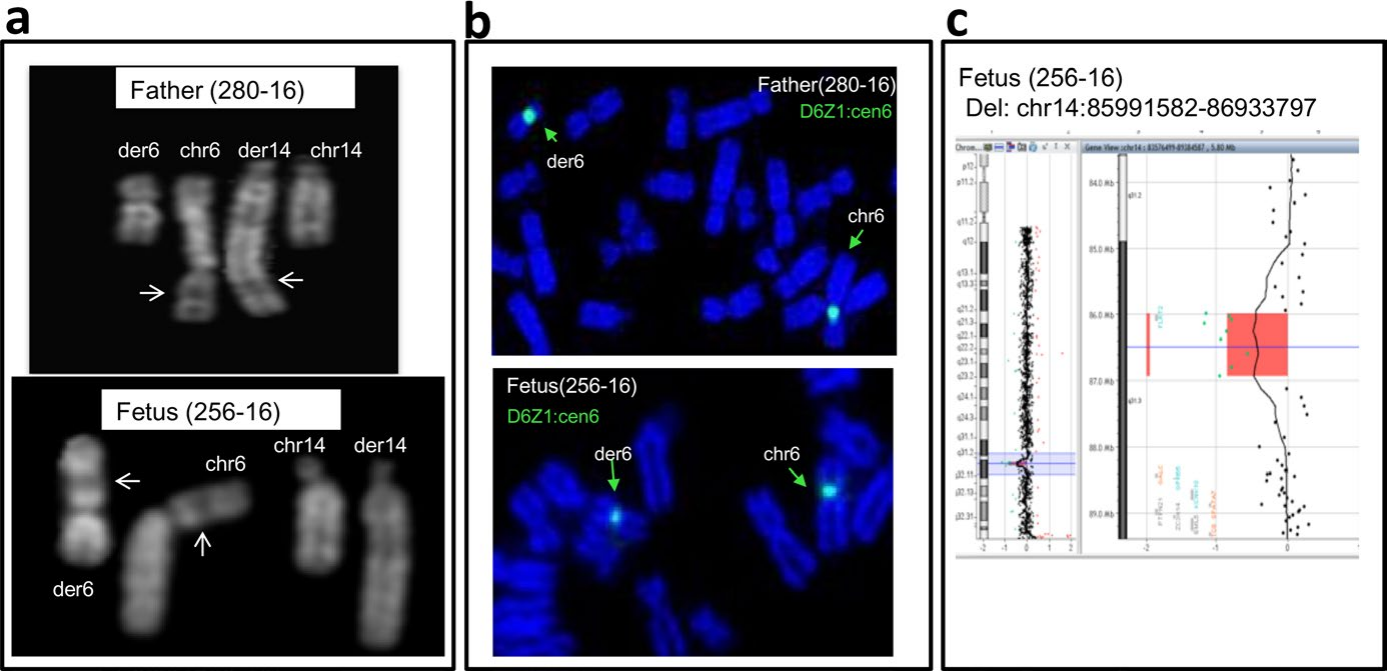
Case 2: rearrangements of chromosome 6 and chromosome 14 in father (280‐16) and fetus (256‐16). (a) Q‐banding revealed different banding patterns of derivative chromosomes 6 (der 6) and 14 (der 14) in father's (280‐16) karyotype compared to der(6) and der(14) of fetus (256‐16): Arrows indicate band 6p21 that appears located at the end of the der(14) in father metaphases (chromosome 6 is upside down to show the regions of homology), while it remains in the der(6) in fetus metaphases. (b) Fluorescence in situ hybridization on DAPI‐stained chromosomes of father (280‐16) and fetus (256‐16) with probe D6Z1 (cen6, green) shows the signal at the end of the paternal der(6) and almost in the middle of the fetus der(6). (c) Array CGH data displaying the de novo ~1Mb deletion (del) on fetus (256‐16)

The proband (256‐16) was a healthy female, born to nonconsanguineous and healthy parents. Her mother underwent amniotic fluid investigation due to anxiety. Karyotype analysis, later on enlarged to parents, revealed that the fetus (256‐16) had a paternally inherited reciprocal translocation t(6:14). Q‐banding on father's metaphases showed that the translocation, although involving the same two chromosomes, resulted in a der(6) and a der(14) with a morphology very different with respect to the two derivates detected in the fetus (Figure 3a). FISH analysis confirmed this diversity (Figure 3b). Additionally, array CGH data showed a de novo 1 Mb deletion at 14q31.3 (chr14: 85991582–86933797) in the fetus's DNA (256‐16) (Figure 3c). Microsatellite analysis by using probes targeting chromosomes 6 and 14 of both fetus and father confirmed the biparental origin of the chromosomes in the fetus (Supporting Information Table  S2).

#### 3.2.2 Whole genome sequencing of father DNA revealed a chromothripsis event

After WGS with an average coverage of 33x, the paternal derivative chromosomes 6 and 14 were reconstructed according to discordant paired reads at the breakpoints detected by NGS data. As a result, chromosome 6 was shattered by seven breakpoints producing eight fragments, named as 6a, 6b, 6x, 6c, 6d, 6e, 6f, and 6g, while chromosome 14 was shattered by three breakpoints producing four segments, named as 14h, 14i, 14l, and 14m (Supporting Information Figure  S5a). Regarding chromosome 6, the rearrangement, as confirmed by Sanger sequencing (Supporting Information Figure  S5b), included (Table 1) the following: (a) inverted orientation of fragment 6 g and 6x within the chromosome 6, (b) translocation of fragment 6f and 6a to the q arm of chromosome 14, and (c) deletion of the fragments 6b and 6c (5.3 kb and 3.7 kb respectively), as identified by decreased coverage (Supporting Information Figure  S6). We were unable to confirm by Sanger sequencing the translocation of 6x, a 350‐bp fragment located in between the deleted 6b and 6c. Chromosome 14 rearrangement involved (a) the inversion of 14i, (b) translocation of 14 m to the 6q, and (c) translocation of fragment 14l to an unrecognized part of the genome (Supporting Information Figure S7). Altogether, we verified and fully characterized five out of seven predicted breakpoint junctions, namely BPJ_6g(−)_6d(+), BPJ_6e(+)_14m(+), BPJ_14h(+)_14i(−), BPJ_14i(−)_6f(+), and BPJ_6f(+)_6a(−) (Table 1, Supporting Information Figure S5b). At the fusion junction of three breakpoints, BPJ_6g(−)_6d(+), BPJ_6e_14m, and BPJ_14h(+)_14i(−), we detected small deletions ranging from 20 bp to 135 bp. In the remaining breakpoint junctions, we observed an insertion of a single bp and microhomology of 3 bp at BPJ_14i(−)_6f(+), and a blunt fusion at BPJ_6f(+)_6a(−) (Table 1, Supporting Information Figure S5).

The fragment 14l of the father, chr14:85953778–86997986, overlapped the same genomic region of the 1 Mb “de novo*”* deletion of the fetus (256‐16) detected by previous array CGH analysis. The sequencing reads on both proximal and distal breakpoints of the fragment 14l were mapped to an unrecognized portion (Supporting Information Figure S7a,b) that we were unable to identify by using NGS data. In order to highlight where fragment 14l was located on father's genome, we designed a custom FISH probe (14q31.1, green) targeting it. FISH analysis performed with the 14q31.1 probe (green) and D6Z1 (cen6, green) revealed that 14l was inserted within chromosome 22p, and not on the der(6) or the der(14) (Supporting Information Figure S7c). Consistently with array CGH data, the fetus (256‐16) did not inherit this 14l fragment.

**Figure 4:**
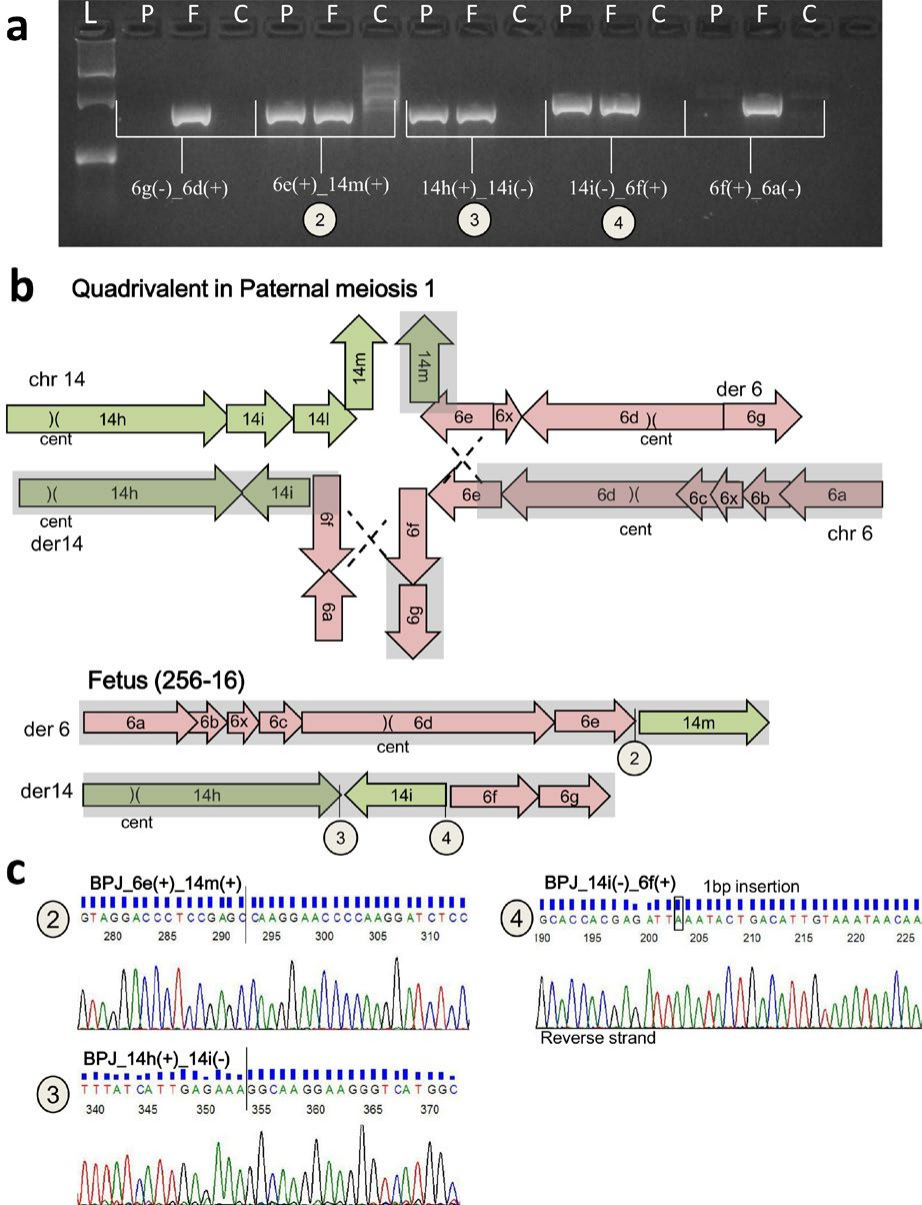
Case 2: Schematic view of the hypothetical recombination event during paternal meiosis I (fragments size not in scale). (a) Breakpoint‐specific PCR for the t(6;14) chromothripsis, performed on fetal (256‐16) and father (280‐16) DNA. P: fetal DNA, F: father, C: control DNA and L: ladder (GelPilot 100 bp plus ladder; Qiagen). The fetus inherited the fusion junctions of 6e(+)_14m(+) (chr6:91762539::chr14:86997986, number 2), 14h(+)_14i(−) (chr14:85427520::chr14:85953778, number 3), 14i(−)_6f(+) (a) (chr14:85427656::chr6:91762553, number 4). (b) Schematic illustration of quadrivalent formed by normal and chromothriptic chromosomes 6 and 14 at the in paternal meiosis I. Numbers 2, 3, and 4 illustrate inherited junctions. (c) Sanger sequencing of fusion junctions 6e(+)_14m(+) (number 2), 14h(+)_14i(−) (number 3), 14i(−)_6f(+) (number 4) in fetal DNA (256‐16)

In order to confirm the paternal origin of the t(6;14) in the fetus, we repeated the breakpoint cloning analysis in the fetal DNA obtained from amniotic sampling (Figure 4a). As a result, the fetus inherited the identical three fusion junctions 6e(+)_14m(+), 14h(+)_14i(−), and 14i(−)_6f(+) confirming that the translocation was the result of the recombination between the paternal chromothriptic chromosomes 6 and 14 (Figure 4b and Figure 4c).

We screened paternal grandparents for the SNPs located at the father‐specific fusion junctions; however, we could not obtain any informative SNP data to highlight the grandparental origin of rearranged chromosomes 6 and 14.

### 3.3 Case 3

#### 3.3.1 Family story

The proband is a 14‐year‐old boy born at 33 weeks of gestation after eventful pregnancy and delivery. He was the only child of unrelated parents, aged 42. His mother had a previous spontaneous abortion at the second month of pregnancy. His weight was 2,000 g, length 45 cm, and OCF 31 cm, all slightly above the 50th centile for preterm infants born at the same gestational age (Villar et al., 2015). Apgar score was 9 at 1 min and 9 at 5 min. He presented a patent ductus arteriosus and hypospadias that were later surgically corrected. Development milestones were delayed: He crawled at 12 months, walked autonomously at 18 months, and started babbling at 12 months, and his language was limited to few words at 18 months. Sphincter control was acquired at 5 years. He attended prescholar nursery, where he was followed by a support teacher and showed difficulties in social interactions. At age of 3.5 years, he was referred to a pediatric neurology service for assessment of global developmental and speech delay. Metabolic workup (urine organic acids, serum and leukocyte lysosomal enzymes, plasma, and urine amino acids) was normal. Audiological and audiometric examination, and auditory‐evoked potentials resulted in the normal range. Due to impairment of motor coordination and speech difficulties, a rehabilitating psychomotor and logopedics therapy was started. At the age of 6.7 years, functional evaluation of the language and neurological observation were performed. A global immaturity was observed, and his emotions were badly controlled: He quickly moved from passivity to provoking behavior when he felt he could not accomplish a given task. Receptive and expressive language was impaired and difficult to understand: Communication was reduced with few words spoken and many mistakes in phoneme production. Social interactions were limited: He continuously searched for his parent attention, his listening was discontinuous, and his answer was often not adequate to the context; the overall results placed the child at the age of about 4 years. At age 12, WPPSI‐IV (Wechsler Preschool and Primary Scale of Intelligence‐Fourth Edition) was administered to the child and placed him within the moderate–severe ID range, with the following index scores: Verbal Comprehension, 62; Perceptual Reasoning, 87; Working Memory, 52; Processing Speed, 69, with a full I.Q, 58. Standard scores of verbal tests were as follows: similarities, 6; vocabulary, 3; and comprehension, 2; nonverbal tests scores were as follows: picture concepts, 7; block design 10; and matrix reasoning, 7. A language questionnaire HBQ (The MacArthur Health and Behavior Questionnaire) placed the child at the age of about 2 years. A discrepancy between language and working competences was noted, and this was mainly referred to his deficit in the working memory area. His reading abilities were poor, corresponding to a first‐grade pupil (6 years). Text comprehension and object manipulation/working abilities were adequate.

**Figure 5:**
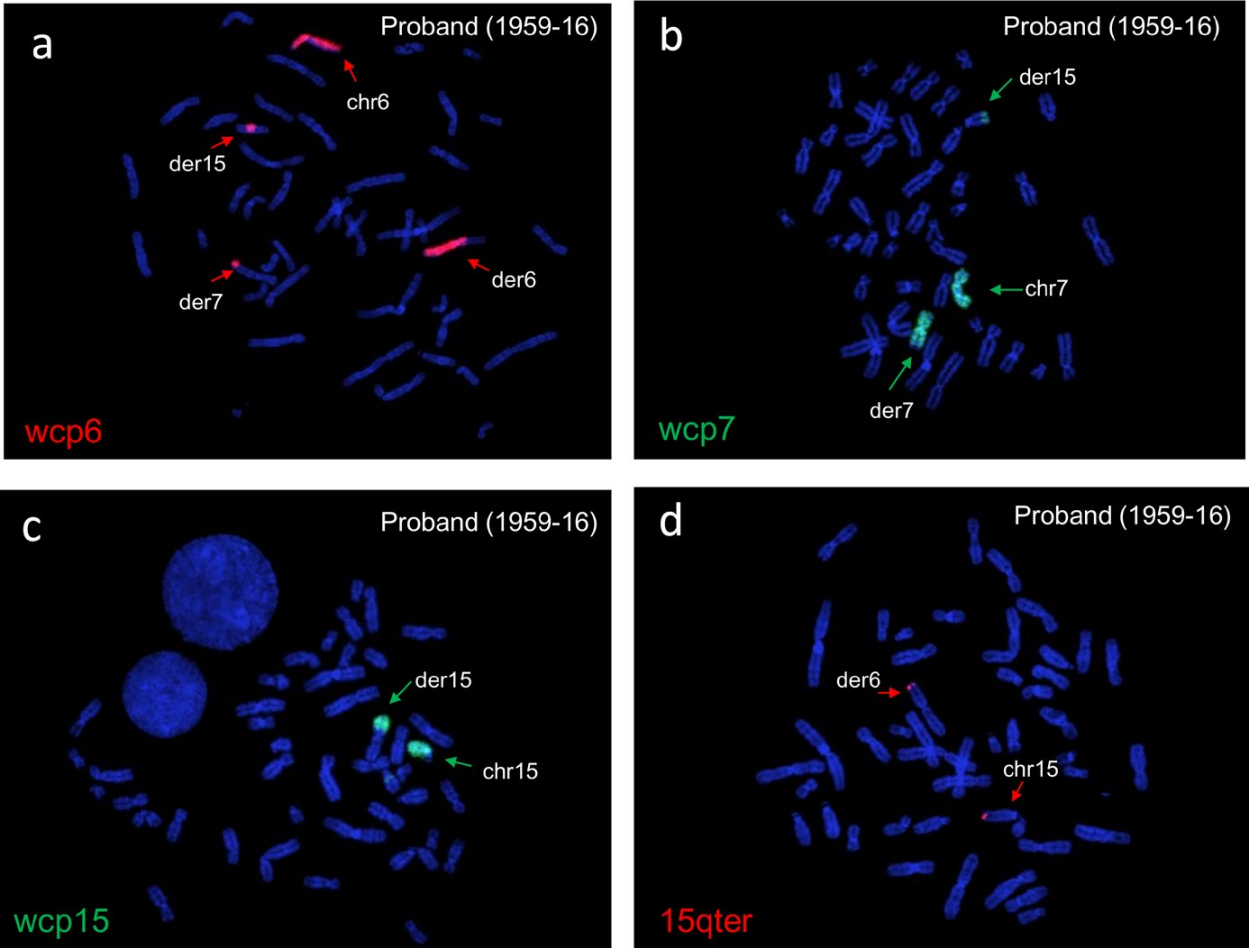
Case 3: Cytogenetics of t(6;7;15) on proband (1959‐16). (a) FISH analysis by WCP of chromosome 6 showed the derivative chromosome 6 and its fragments at distal 7q and the middle of 15q, (b) WCP of chromosome 7 showed derivative chromosome 7 and part of it at the distal 15q. (c and d) WCP of chromosome 15 and FISH analysis performed with chromosome 15 telomeric probe (15qter, red) painted both the derivative chromosome 15 and distal p portion of the derivative chromosome 6. FISH: fluorescence in situ hybridization

Conventional cytogenetics, later on extended to the parents, revealed a maternally inherited complex translocation between chromosomes 6, 7, and 15, including a 116 Mb pericentric inversion of chromosome 6 (Figure 5). The proband's karyotype was defined as: 46,XY,t(6;7;15)(15qter‐>15q15::6q21‐>6qter;7pter‐>7q32::6p23‐>6pter;15pter‐>15q15::6p21.3‐>6p23::7q32‐>7qter)mat.

The mother's developmental milestones were almost in the normal range: She walked autonomously at 18 months, and first words were spoken at 11 months. She presents strabismus and a very irregular dentition. She had difficulties during her schooling, but neither attended a school for special needs nor had support teachers. She was not able to obtain a high school diploma. She is now working as a homecare provider and has good social integration. Cognitive ability assessments have never been performed.

#### 3.3.2 Whole genome sequencing results in mother's and proband's DNA

**Figure 6:**
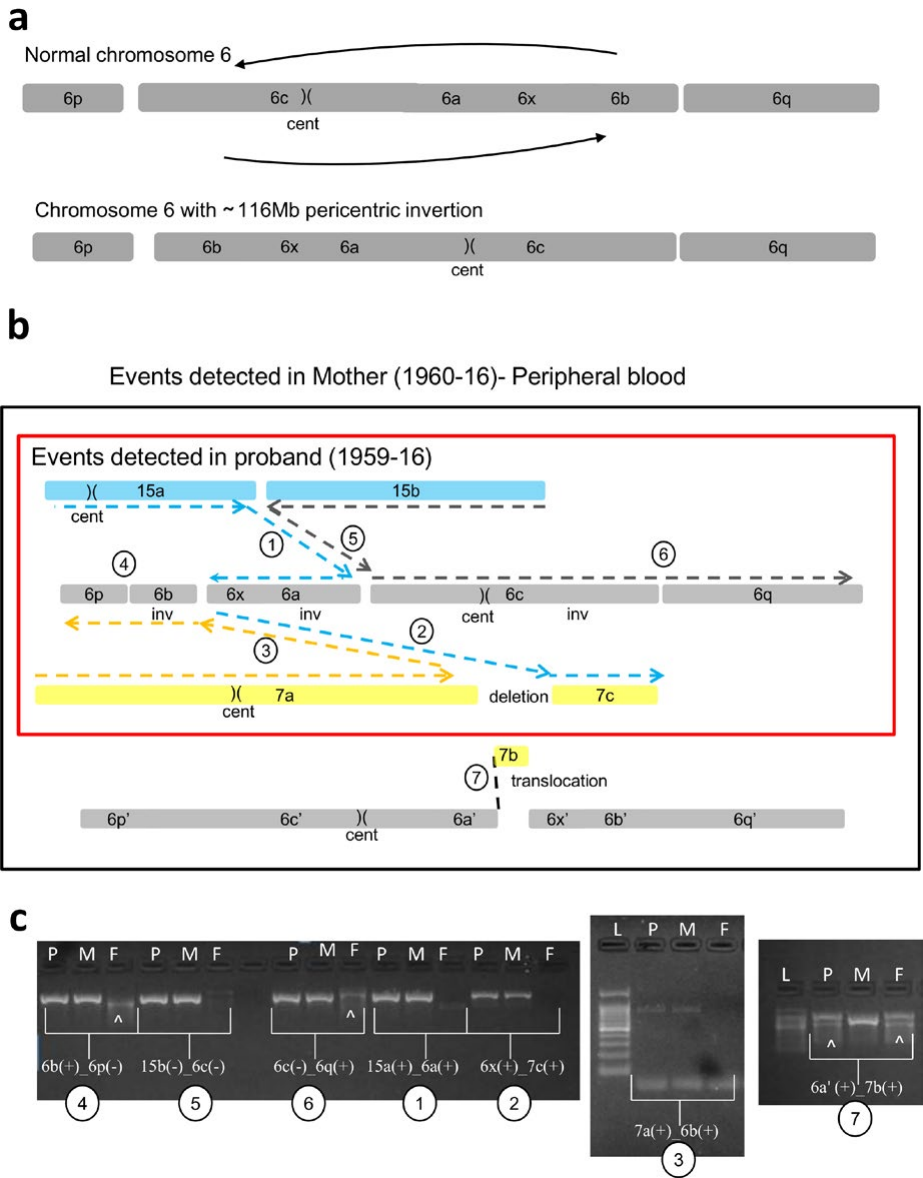
Case 3: Schematic view of t(6;7;15) chromothripsis of mother (1960‐16) and proband (1959‐16) (fragment size not in scale). (a) Shattering of chromosome 6 as a result of t(6;7;15) produced the fragments 6p, 6b, 6x, 6a, 6c, and 6q, altogether involving a 116 Mb pericentric inversion of chromosome 6 (cent: centromere). (b) Rearrangements of t(6;7;15) in mother's peripheral blood DNA involving mother‐specific 6a′‐7b breakpoint junction. Reconstruction of derivative chromosomes is illustrated with dashed lines, blue for derivative chromosome 15, gray for derivative chromosome 6, and yellow for derivative chromosome 7. Each fusion junctions are numbered from 1 to 7. The rearrangement detected in the proband is shown in a red rectangle. (c) Breakpoint‐specific PCR for the chromothripsis t(6;7;15) performed on proband (1959‐16), mother (1960‐16), and father (1961‐16). Case‐specific fusion junctions 6b(+)_6p(−) (number 4), 15b(−)_6c(−) (number 5), 6c(−)_6q(+) (number 6), 15a(+)_6a(+) (number 1), 6x(+)_7c(+) (number 2), and 7a(+)_6b(+) (number 3) are verified by the amplification of around 800 bp target size both in the mother and proband, but not in the father as expected. Fusion junction 6a′(+)_7b(+) (number 7) was verified only in the mother's DNA. The data proved that proband inherited all the breakpoints of t(6;7;15) except the fusion junction 6a′(+)_7b(+) (c). P: proband, M: mother, F: father, L: Ladder (GelPilot 100 bp plus ladder; Qiagen), (^): signifies nonspecific amplification

The average coverage of WGS experiments was 31x in the mother DNA (1960‐16) and 39x in proband's one (1959‐16). In proband's DNA (1959‐16), we detected a total of seven breakpoints: four on chromosome 6, involving those of the pericentric inversion, producing five fragments (6a, 6b, 6c, 6p, and 6q) (Figure 6a), two on chromosome 7 producing three fragments (7a, 7b, and 7c), and one on chromosome 15 producing two fragments (15a and 15b; Figure 6b, Table 1, Supporting Information Figure S8a). In the mother, in addition to the breakpoints transmitted to the proband, WGS analysis revealed a further breakpoint on chromosome 6 indicating a fusion junction of chromosomes 6 and 7 (6a‐7b fusion junction, chr6:111343917::chr7:132591776; Table 1, Supporting Information Figure S9). Notably, this 6a‐7b breakpoint was absent in the proband's genome. Additionally, fragment 7b, of 4,297 kb (chr7:132591756–132596053), was deleted in the proband as suggested by decreased coverage of the region, while it was in balanced state in the mother (Supporting Information Figure S10). Overall, these findings, more specifically the deletion of fragment 7b and the absence of 6a‐7b junction in proband's genome, allowed to hypothesize that in the mother, this 7b fragment was translocated to the “normal” chromosome 6 (Figure 6b), suggesting a postzygotic origin of the complex rearrangement in the mother. In this model, in addition to the seven breakpoints as detected in the proband, the mother had two further breakpoints on the homologous chromosome 6 producing three fragments 6a′, 6x′, and 6b′ (apostrophe on the fragments was used to indicate the homolog pair of the chromosome fragment 6).

Polymerase chain reaction and Sanger sequencing were performed to verify previous data (Supporting Information Figure S8b). As a result, the breakpoint junctions of t(6;7;15), BPJ_15a(+)_6a(+), BPJ_6x(+)_7c(+), BPJ_7a(+)_6b(+), BPJ_6b(+)_6p(−), BPJ_15b(−)_6c(−), and BPJ_6(−)_6q(+) were confirmed both in proband (1959‐16) and in mother (1960‐16; Figure 6c), leading to final interpretation of the rearrangement different from the original one: 46,XY,t(6;7;15)(15qter‐>15q15.1::6q14.2‐>6p25.1::6q22.31‐>6qter;7pter‐>7q32.3::6q21‐>6q22.31::6p25.1‐>6pter;15pter‐>15q15.1::6q14.2‐>6q21::7q32.3‐>7qter). As concordant with the NGS data, breakpoint junction between 6a′ and 7b, BPJ_6a′(+)_7b(+), was present only in the mother supporting the reconstruction model of t(6;7;15). Sequence features at breakpoint junctions involved microhomology (four out of seven), small deletions up to 100 bp (four out of seven), and 6 bp microduplication (one out of seven; Table 1; Supporting Information Figure S8b). In order to test whether the mother was mosaic for the breakpoints of t(6;7;15), we performed PCR analysis of all t(6;7;15) breakpoints on her saliva‐derived DNA. As a result, six of the fusion junctions including the one between 6a′ and 7b, BPJ_6a′(+)_7b(+), were present also in the saliva, while unexpectedly the fusion junction between 7a and 6b, BPJ_7a(+)_6b(+), was absent (Supporting Information Figure S11), suggesting that the complex translocation t(6;7;15) underwent small additional rearrangements in a tissue‐specific way. As repeat elements upstream to the 6a′‐7b breakpoint at chromosomes 6 impaired PCR cloning and SNP screening, we could not confirm the involvement of both chromosome 6 homologs. Absence of loss of heterozygosity on chromosomes 6, 7, and 15, as demonstrated by SNP‐CGH array performed on proband, excluded uniparental disomy (data not shown). We screened maternal grandparents for the SNPs located at the t(6;7;15) fusion junctions, and we obtained a single informative SNP located at the inversion junction at chromosome 6, BPJ_6b(+)_6p(−), suggesting the grandpaternal origin of rearranged chromosome 6 (Supporting Information Figure S4).

### 3.4 Genes and Sequence elements at the breakpoints of chromothripsis

In all the three cases, chromothripsis breaks occurred either within intergenic regions or within introns but never within exons, according to UCSC Genome Browser GRCh37/hg19 (Table 2, Supporting Information Table S3). In case 1, *ROPN1B, NAALADL2* (OMIM 608806), and *TF* (OMIM 190000) were disrupted within intron 4 (NM_001308313), intron 1 (NM_207015), and intron 5 (NM_001063), respectively. In case 2, *OPRM* (OMIM 600018) and *RNGTT* (OMIM 603512) were disrupted within intron 3 (NM_000914) and intron 13 (NM_003800). In case 3, four disrupted protein‐coding genes, namely *CASC5* (OMIM 609173), *RPF2* (OMIM 614443), *CHCHD3* (OMIM 613748), and *CLVS2* (OMIM 616945), were respectively broken at intron 14 (NM_170589), intron 8 (NM_032194), intron 4 (NM_001317177), and intron 4 (NM_001010852). In no case, fusion genes have been created.

**Table 2:** Genes located at the breakpoint junctions

Fusion junction	Chromosomal position	Genes	Fusion junctions at the gene level
Case 1			
BPJ_3a(+)_3b(−)	chr3:125695543::chr3:133464556	*ROPN1B‐TF*	NM_001308313.1:c.235‐304::NM_001063.3:c.‐729
BPJ_3b(−)_3e(−)	chr3:125695544::chr3:174480425	*ROPN1B*	NM_001308313.1:c.235‐303::chr3:g.174480425
BPJ_3e(−)_3g(−)	chr3:169592489::chr3:174724061	*NAALADL2*	chr3:g.169592489::NM_207015.2:c.44‐90519
BPJ_3d(+)_3c(+)	chr3:169592488::chr3:133464557	*TF*	chr3:g.169592488::NM_001063.3:c.‐728
BPJ_3c(+)_3h(+)	chr3:143880432::chr3:174724062	*NAALADL2*	chr3:g.143880432::NM_207015.2:c.44‐90518
Case 2			
BPJ_6g(−)_6d(+)	chr6:154434404::chr6:48394698	*OPRM1*	NM_001145279.3:c1444‐5415_5434del20::chr6:g.48394698
BPJ_6f(+)_6a(+)	chr6:154434383::chr6:48385249	*OPRM1*	NM_001145279.3:c1444‐5435::chr6:g.48385249
Case 3			
BPJ_15a(+)_6a(+)	chr15:40926862::chr6:84684894	*CASC5*	NM_170589.4:c.5760+5293::chr6:g.84684894
BPJ_6x(+)_7c(+)	chr6:111349988::chr7:132596053	*CHCHD3*	chr6:g.111349988::NM_001317177.1:c.370‐24305
BPJ_7a(+)_6b(+)	chr7:132591756::chr6:111349990	*CHCHD3*	NM_001317177.1:c.370‐20008::chr6:g.111349990
BPJ_6b(+)_6p(−)	chr6:123371910::chr6:6793991	*CLVS2*	NM_001010852.3:c.675+2033::chr6:g.6793991
BPJ_15b(−)_6c(−)	chr15:40926874::chr6:84684793	*CASC5*	NM_170589.4:c.5760+5294_5304del11::chr6:g.84684793
BPJ_6c(−)_6q(+)	chr6:6793992::chr6:123371911	*CLVS2*	chr6:g.6793992::NM_001010852.3:c.675+2034
BPJ_6a′(+)_7b	chr6:111343917::chr7:132591776	*RPF2‐CHCHD3*	NM_032194.2:c.597‐1469::NM_001317177.1:c.370‐20028_20009del19

Note

Fusion junctions involving gene disruption are listed. Human Genome Variation Society (HGVS; Taschner & Dunnen, 2011) nomenclature is used to describe fusion junctions at the gene level.

We examined the overall distribution of repeat elements (UCSC RepeatMasker) nearby the breakage sites of rearranged chromosomes (Supporting Information Table S3). In case 1, four out of nine breakpoints, namely the two deletion breakpoints of chromosome 8 and two breakpoints (3d‐3e and 3g‐3h) at chromosome 3, were located at long interspersed elements (LINE). In case 2, six out of 10 breakpoints were enriched with a varied type of repeat elements: long terminal repeats (LTR), Alu elements, DNA repeats, and LINE‐1. In case 3, three out of eight breakpoints were located at repeat elements; LTR, LINE 1, and LINE 2.

## 4 DISCUSSION

We illustrate here three cases, two regarding persons with a disease phenotype and one a fetus that appeared normal at ultrasonography, all carrying an unbalanced genomic rearrangement derived by a parent having structural variation(s) involving the same chromosome(s) that are abnormal in the proband but, at least in cases 1 and 2, with a totally unexpected configuration as revealed by WGS. Similar findings have already been reported (de Pagter et al., 2015), although in these two cases the unpredictable genomic complexity we detected in the carrier parents, indeed, challenged the current rule of thumbs, we use to define the risk of recurrence of genomic imbalances in subsequent pregnancies. Also in case 3, the only one in which conventional cytogenetics indicated that both the proband and his mother carried a complex rearrangement, WGS highlighted a higher complexity but in line with what is currently known.

### 4.1 Case 1

The proband, a male child essentially showing psychomotor delay, resulted normal at conventional cytogenetics, whereas the array CGH in the trio detected two de novo imbalances at 3q. FISH investigation showed that the duplication consisted in an intrachromosomal insertion. Moreover, FISH in mother's metaphases showed a possible paracentric inversion.

Constitutional rearrangements consisting in a terminal deletion preceded by a normal region of variable size and a more proximal duplication are not rare. They are de novo events, resulting from an intermediate dicentric chromosome originated by either breakage–fusion–bridge cycle mechanism (Hermetz et al., 2014) or nonallelic homologous recombination (NAHR), the latter one favoured by the presence of a polymorphic inversion within the segmental duplications at the basis of the NAHR (Zuffardi, Bonaglia, Ciccone, & Giorda, 2009). Proband's 3q rearrangement did not appear to be the consequence of the breakage of a dicentric chromosome. Indeed, both the deletion and the duplication were interstitial and the maternal inversion was much larger than that associated with an intermediate dicentric (Hermetz et al., 2014), it was a de novo one and not reported as a polymorphism, thus likely excluding a NAHR‐mediated event. All these data alerted us on a presumably catastrophic event on the maternal 3q with the proband's chromosome 3 possibly being a recombinant one. WGS on maternal and proband DNA substantiated this hypothesis showing that the mother carried a balanced 3q with seven breakpoints within 49 Mb at 3q21‐q24.3. The reconstruction of the shattered fragments, later confirmed by breakpoint junctions PCR, revealed that the novel chromosome 3 lacked a 3.3‐kb fragment, fragment 3f at 3q26.31, that was transposed to 8p12, generating a submicroscopic insertional translocation, whereas the receiving chromosome 8 resulted to be deleted for 6.8 kb at the region of insertion. The signature pattern of the breakpoints showed repair‐based mechanisms such as NHEJ and microhomology‐mediated processes (Chiang et al., 2012; Kloosterman et al., 2012; Kloosterman, Guryev, et al., 2011; Kloosterman, Hoogstraat, et al., 2011), whereas the absence of copy‐number gains excluded replication‐related pathways.

Altogether, this complex rearrangement fits with a chromothripsis‐like event both for the shattering of a large portion of an individual chromosome into many fragments (Ly & Cleveland, 2017) and for the presence of an insertional translocation associated with a small deletion of the receiving chromosome next to the insertion site, as recently reported (Gu et al., 2016; Kato et al., 2017). The reconstruction of maternal and proband's rearrangement by WGS clearly indicated that proband's imbalance was the result of a meiotic recombination (Figure 2) involving chromosome 3 only and leaving chromosome 8 intact. In future pregnancies, careful evaluation of fetal DNA by high‐resolution array CGH should take into consideration possible imbalances concerning chromosome 8 as well.

### 4.2 Case 2

In this case, cytogenetics on amniotic fluid cells was requested for maternal anxiety. The finding of a reciprocal translocation activated the extension of the analysis to the healthy parents in order to clarify whether it was inherited and thus presumably benign or whether, in the case of a de novo rearrangement, molecular investigations were necessary. Furthermore, the involvement of two chromosomes, 6 and 14, both containing imprinted genes, suggested investigating whether they were of biparental origin (Supporting Information Table S2). The presence in the father of a translocation between chromosomes 6 and 14 but with the two derivatives having a morphology very different from that of the proband alerted us on the possibility that the father was carrier of a chromothriptic event of which proband's chromosomes 6 and 14 were the recombinant ones. The array CGH in the trio revealed a de novo 1 Mb deletion at 14q31.3 in the fetal DNA and WGS in the paternal DNA confirmed that chromosomes 6 and 14 underwent extensive shattering followed by intra‐ and interchromosomal stitching. This reconstruction showed that fragment 14l, deleted in the fetus (Figure 3c), was translocated to an unrecognized part of the genome. Later FISH investigation on paternal metaphases showed that it was inserted within 22p, a region that, as all short arms of the acrocentric chromosomes, is missing from the current human genome draft (McStay, 2016). Altogether, case 2, similar to case 1, fits with a chromothripsis‐like event leading in the father to an apparently reciprocal translocation involving chromosomes 6 and 14. Sequence analysis on the breakpoints showed less precisely fused break‐end with three out of five breakpoints that were shortened by small deletions as frequently observed in translocation formation in mammalian cells (Simsek & Jasin, 2014; Weinstock, Elliott, & Jasin, 2006). The remaining junctions displayed repair‐based mechanisms as NHEJ and microhomology‐mediated processes. As in case 1, the catastrophic event was accompanied by an insertional translocation leading to the insertion of a fragment of one of the two chromothripsed chromosomes (fragment 14l) within a nonchromothripsed one, namely 22p. The novel configuration of proband's translocation was the result of the recombination between the paternal chromothriptic chromosomes 6 and 14 (Figure 4b,c).

Similar instances of inherited simple reciprocal translocations involving the same two chromosomes in parent and proband but at different breakpoints had not been previously reported. The 1 Mb 14q deletion detected in amniotic fluid, which was at first interpreted as de novo, has been considered as likely benign essentially due to the absence of known OMIM genes whose haploinsufficiency was disease‐associated. Moreover, in the only DECIPHER patient, 288,747, having a comparable size deletion, the inheritance is unknown making doubtful its pathogenicity. Thus, taking also into consideration that the fetus was normal at ultrasounds, parents decided to continue the pregnancy. The female child, presently more than 2 years old, is healthy and shows a psychomotor development fully adequate for her age, suggesting that the 14q CNV was really benign. In future pregnancies, in the case of a normal fetal karyotype, array CGH is indicated to exclude any submicroscopic deletion or duplication involving chromosomes 6, 14, and 22. In case of a fetus carrying the reciprocal translocation, in addition to array CGH, cloning of chromothripsis breakpoints should be applied to exclude overlooking small deletion/duplications.

### 4.3 Case 3

In this case, the four‐year‐old proband was referred because of psychomotor delay, while the mother was healthy although achieved a low educational level with respect to the socioeconomical level of the family. None of the disrupted genes was associated with a known autosomal dominant disease. Regarding the chromothripsis as characterized by WGS, the only genotypic difference between mother and son concerns the 4.2‐kb region belonging to fragment 7b that is deleted in the proband, while it appeared to be insertionally translocated within the nonchromothripsed chromosome 6 in the mother. Thereafter, we may assume that the condition of mosaicism of the complex rearrangement in the mother as indirectly demonstrated by breakpoint cloning in two different DNA samples is responsible for the psychomotor delay evident in the child. Altogether, these findings suggest that the shattering of chromosomes 6, 7, and 15 occurred in one cell of the early embryo while the stitching of their fragments either did not happen in a single moment or was subjected to subsequent rearrangements in the different cell lines, as reported in tumors (Collins et al., 2017). We should note that, due to the absence of NGS analysis on saliva DNA, reconstruction models other than the one proposed in this study would be also possible for t(6;7;15) in the mother's DNA.

### 4.4 Chromothripsis and insertional translocations

It is worthwhile to note that in all three cases, the chromothriptic event was associated with an insertional translocation involving an additional chromosome. Several examples of chromothriptic‐like events associated with insertional translocation have been recently reported in cases with multiple CNVs (Gu et al., 2016; Kato et al., 2017; Kurtas et al., 2018). Taking into consideration that depending on the sequencing resolution some insertional translocations may escape detection, these events are probably the rule rather than the exception (Slamova et al., 2018). This finding, beyond clarifying the mechanisms of readjustment of broken chromosomes, indicates that insertions should not be anymore considered simple three chromosome breakage events (Weckselblatt & Rudd, 2015), suggesting the opportunity to investigate the entire genome whenever a translocation insertion is evidenced, both if detected in the parent of a proband carrying an apparently de novo CNV or directly in the proband. Indeed, this novel scenario of insertional translocations hiding chromothripsis‐like rearrangements leads to a reproductive risk much higher than previously estimated for the balanced carriers (Nowakowska et al., 2012).

### 4.5 Chromothripsis: Constitutional versus mosaics events

In the healthy parents of cases 1 and 2, we did not find any evidence for the de novo chromothriptic event being a mosaic, at least as we could judge by FISH analysis and breakpoint investigations in the single available tissue. We may assume either that the rearrangement was inherited as such or that the rearrangement occurred in an early embryo cell, being then selected with respect to the normal cells in most tissues. While in the father of case 2 we were unable to highlight the parental origin, in the mother of case 1 the event was of grandmaternal origin (Supporting Information Figure S4), which is in contrast to most de novo chromothriptic events that are of preferential paternal origin (Collins et al., 2017; Grossmann et al., 2010; Kloosterman, Guryev, et al., 2011; Kloosterman, Hoogstraat, et al., 2011). In contrast, in case 3 the finding of the insertion of one fragment coming from the chromothriptic 7 within the normal chromosome 6 and not within its chromothriptic homolog pointed to a postzygotic rearrangement. Accordingly, the three rearranged chromosomes might have a different parental origin. Unfortunately, we were able to detect a single informative SNP that indicated that the chromothripsed 6 was of grandpaternal origin. Thus, we could not discriminate whether the entire complex rearrangement was postzygotic indeed or occurred at the paternal gametogenesis with following postzygotic readjustments of the original configuration, or even whether the inverted chromosome 6, formed at the paternal gametogenesis, initiated a massive genome reorganization in one early embryo cell, followed by minimal readjustments in the different tissues. Indeed, the finding that in case 3 the breakpoint junction 7a(+)_6b(+) was present in mother's blood but not in her saliva indicated that the (three) massively broken chromosomes underwent a series of postzygotic repairs resulting in small differences of the same chromothriptic event in the different tissues. This observation parallels what has been documented in cancer cells where massive genome reorganization, whichever the related mechanisms and clinical outcome, indeed requires evolutionary selection processes to pick up winning genomes that may be different in the different tissues (Behjati et al., 2017; Ye, Liu, & Heng, 2018).

### 4.6 Genome architecture at rearrangements’ breakpoints

According to the alleged role of retrotransposons in chromothripsis (Nazaryan‐Petersen et al., 2016), we checked for all repeat elements at the distal and proximal sites of the breakpoints. In cases 2 and 3, we detected a variety of repeats without enrichment of a single family. In case 1, we observed enrichment of only LINE elements at four breakpoints without any signature of Alu‐mediated L1 activity, thus making our data incompatible with the model of local DNA shattering driven by L1 endonuclease activity at regions, which have been brought in close proximity by Alu‐mediated chromosome looping (Nazaryan‐Petersen et al., 2016).

## 5 CONCLUSION

The study demonstrates how it is not always obvious to understand when a genomic imbalance can in fact be much more complex than it appears from the conventional and/or molecular karyotype and, above all, when it can hide the presence of a cryptic rearrangement in a healthy parent, in turn leading to a high risk of novel genomic imbalances in subsequent pregnancies. All three cases confirm that balanced parental chromothripsis may not be balanced at molecular level (Gu et al., 2016). Of note, in all the three cases, the catastrophic event involving one, two, and three chromosomes respectively was associated with the insertion of one of the shattered fragment into an additional chromosome, as already reported by others (De Gregori et al., 2007; Feenstra et al., 2011; Gribble et al., 2005; Schluth‐Bolard et al., 2009). This finding indeed changes our way of approaching laboratory investigation and genetic counseling once an insertion is detected.

We also provide other examples confirming that fully healthy persons, as it is the case for the parents in families 1 and 2, notably the latter one being ascertained fortuitously, may be carrier of apparently germline catastrophic events, showing on the one hand that germline chromothripsis‐like events without feasible phenotypic effects are more frequent than so far estimated by cytogenetics and chromosomal microarray and on the other one that our genome may tolerate without evident clinical consequences even rearrangements that interrupt TADs (see Supporting Information Appendix S1).

Finally, our study stresses that the use of WGS might be considered as first‐tier investigation in clinical cytogenetics. Low‐pass whole‐genome sequencing appears so far the most convenient procedure, although debate is still ongoing (Cretu Stancu et al., 2017; Dong et al., 2016).

## CONFLICT OF INTEREST

The authors declare no conflict of interest.
