# Supplementary material for: Insertional translocation involving an additional nonchromothriptic chromosome in constitutional chromothripsis: Rule or exception?
Source: Mol Genet Genomic Med. 2018 Dec 18;7(2):e00496. doi: 10.1002/mgg3.496 (PMC6393660; doi:10.1002/mgg3.496)
Supplement: Supplementary file 1 [file MGG3-7-na-s001.docx]

**Supplementary Figures**

**Supplementary Figure S1**

**
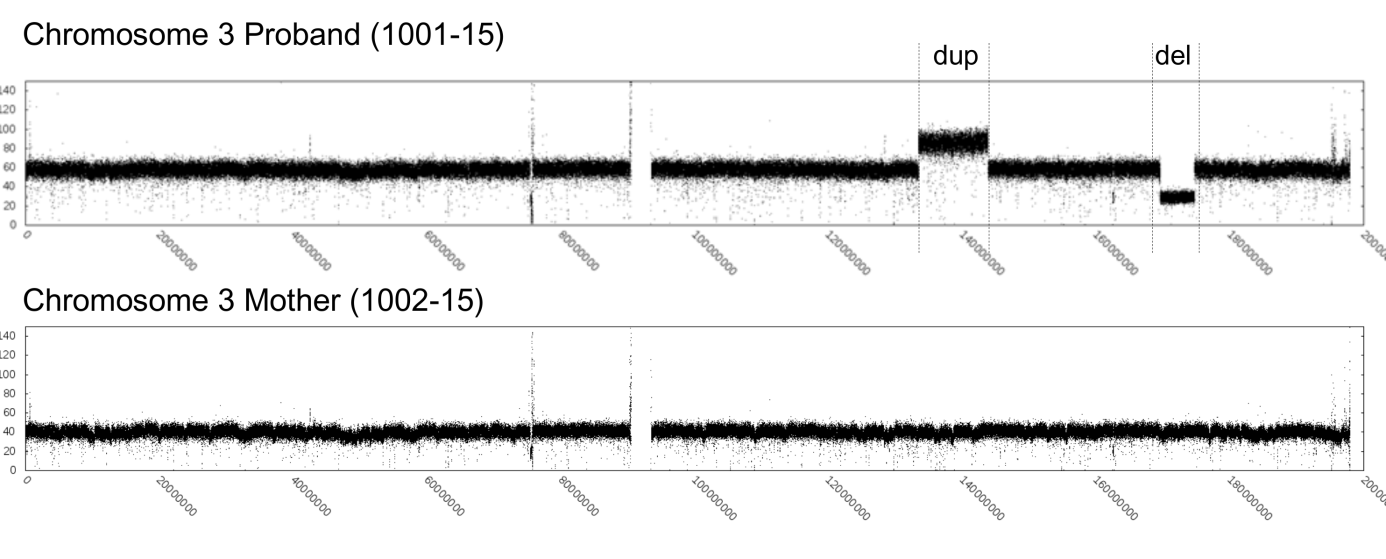
**Case 1. Coverage plot analysis of whole chromosome 3, obtained by paired-end WGS, confirmed duplicated (dup, Chr3:133466557-143862852) and deleted portions (del, Chr3:169599118-174713426) of proband (1001-15) and balanced state of mother (1002-15). The average coverage of whole chromosome 3 was 57.6x, while the average coverage of duplicated and deleted sites were calculated as 85.09x and 29.6x, respectively.

**Supplementary Figure S2**

**
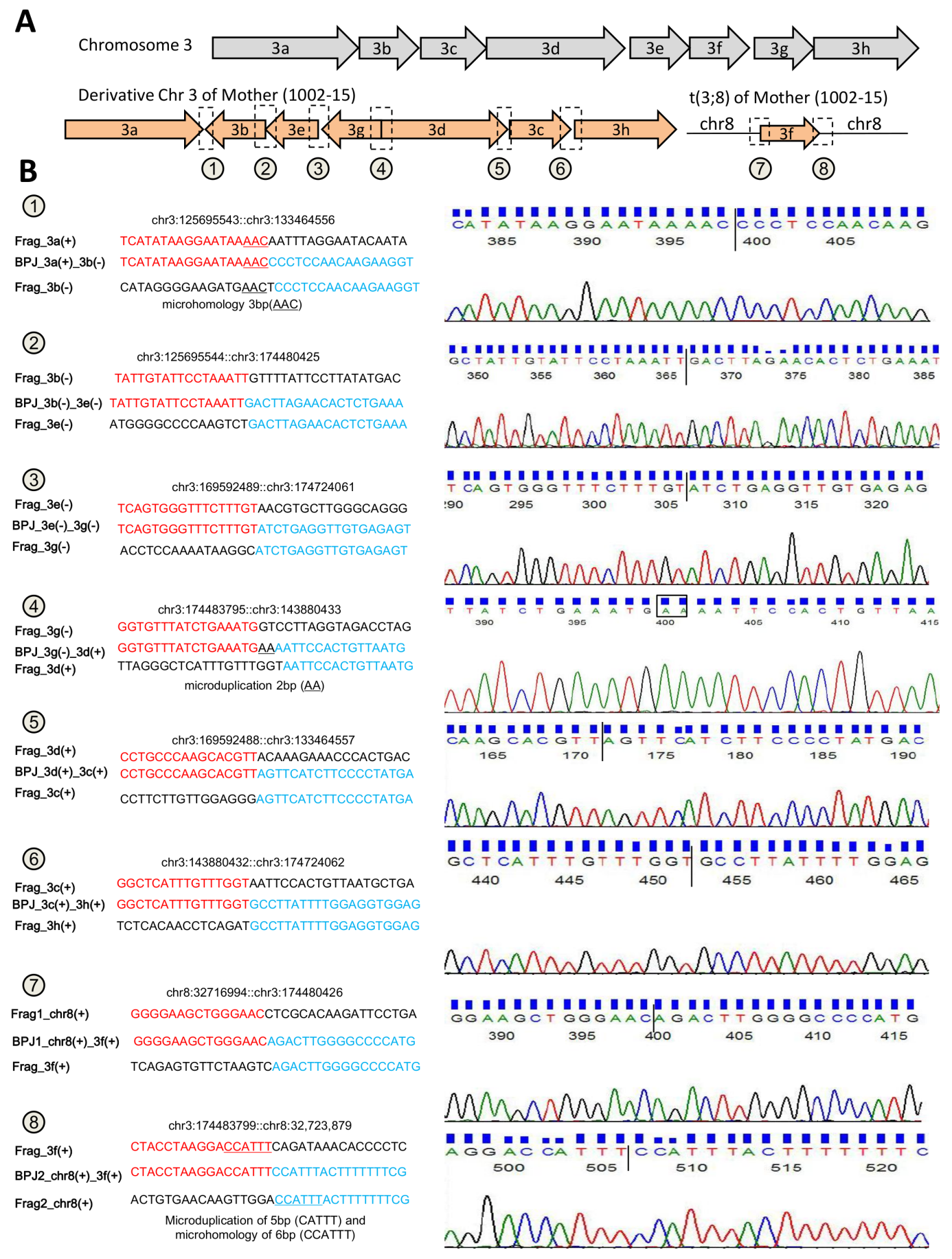
**

Case 1. A: schematic representation of the original order of the shattered fragments (grey) and their novel orientation in derivative chromosome 3q (yellow) of the mother (2002-15, case 1). Chromosomes are represented with the centromeric portion on the left and the long arm telomere on the right. Inverted fragments, 3b, 3e and 3g, are indicated with left arrowheads. Insertion of the fragment 3f within chromosome 8p (short arm telomere on the left, centromere on the right) is shown. Fusion junctions, indicated by dashed rectangles, are numbered from 1 to 8 (numbers in the circle).

B: Sanger validation of each breakpoint junction of the shattered fragments and their reference sequences prior to the chromothripsis (red and blue) are shown. The inverted fragments are indicated with minus (-) sign. Microduplication and microhomology at the breakpoint junctions are underlined in the text. The breakpoints’ signatures indicated NHEJ mechanisms with blunt fusions at five junctions: 2, 3, 5, 6, 7, microduplicationsat two junctions: 4, 8 and microhomologies at two junctions: 1, 8. The junction between chr3 and chr8 translocation, BPJ2_3f(+)_chr8: fusion junction 8, had a different signature, with the involvement of both microhomology and microduplication (5bp). As demonstrated by Sanger sequencing, there is a 5bp sequence overlapping, 5'-CATTT-3' at frag_3f(+) and 5'-GTAAA-3' frag_3g(-), resulting in a microduplication of chr3:174,483,795-chr3:174,483,799, possibly due a sticky ended double stranded break between frag_3f and frag_3g, where 5-base 3′-overhang (5'-CATTT-3') was generated. Moreover, 6bps (5'-CCATTT-3') at frag_3f(+) have a sequence homology at the breakpoint junction of frag2_chr8(+), suggesting the involvement of microhomology mediated mechanism at BPJ2_3f(+)_chr8.

**Supplementary Figure S3**

**
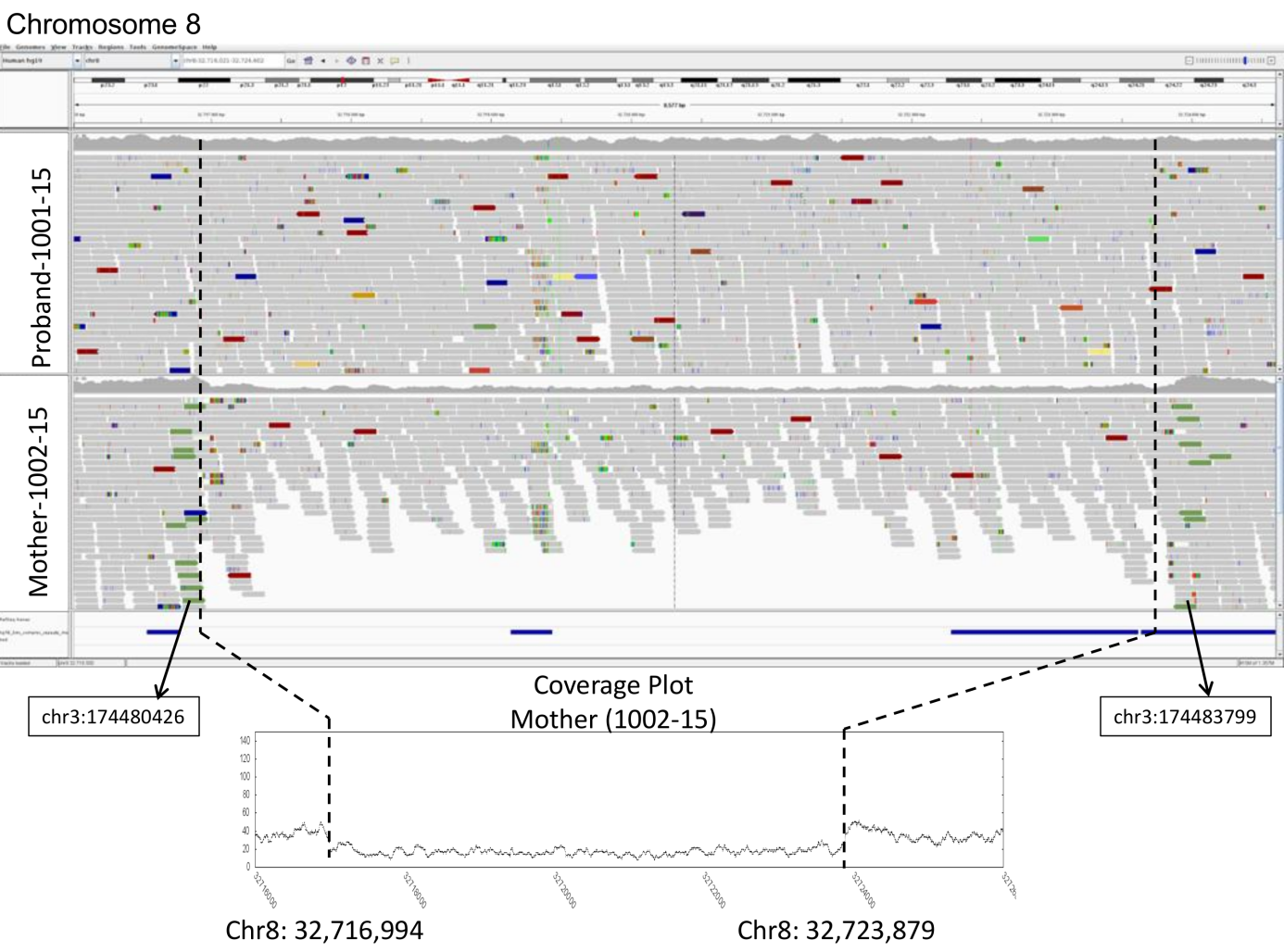
**

Case 1. IGV illustration and coverage plot indicate the heterozygous 6,8kb deletion at chr8:32716994-32723879. The deletion was demonstrated by decreased average coverage 23x in the mother (1002-15), while the average coverage of whole chromosome 8 was 40.2x. The same region in the proband (1001-15) was in balanced state with the average coverage of 57.4x, while the calculated average coverage of whole chromosome 8 was 58.3x. The discordant reads (green) at both left and right side of the deleted portion mapped to chr3:174480426 and chr3:174483799 respectively, indicating the insertional translocation of fragment 3f into the chromosome 8.

**Supplementary Figure S4**

**
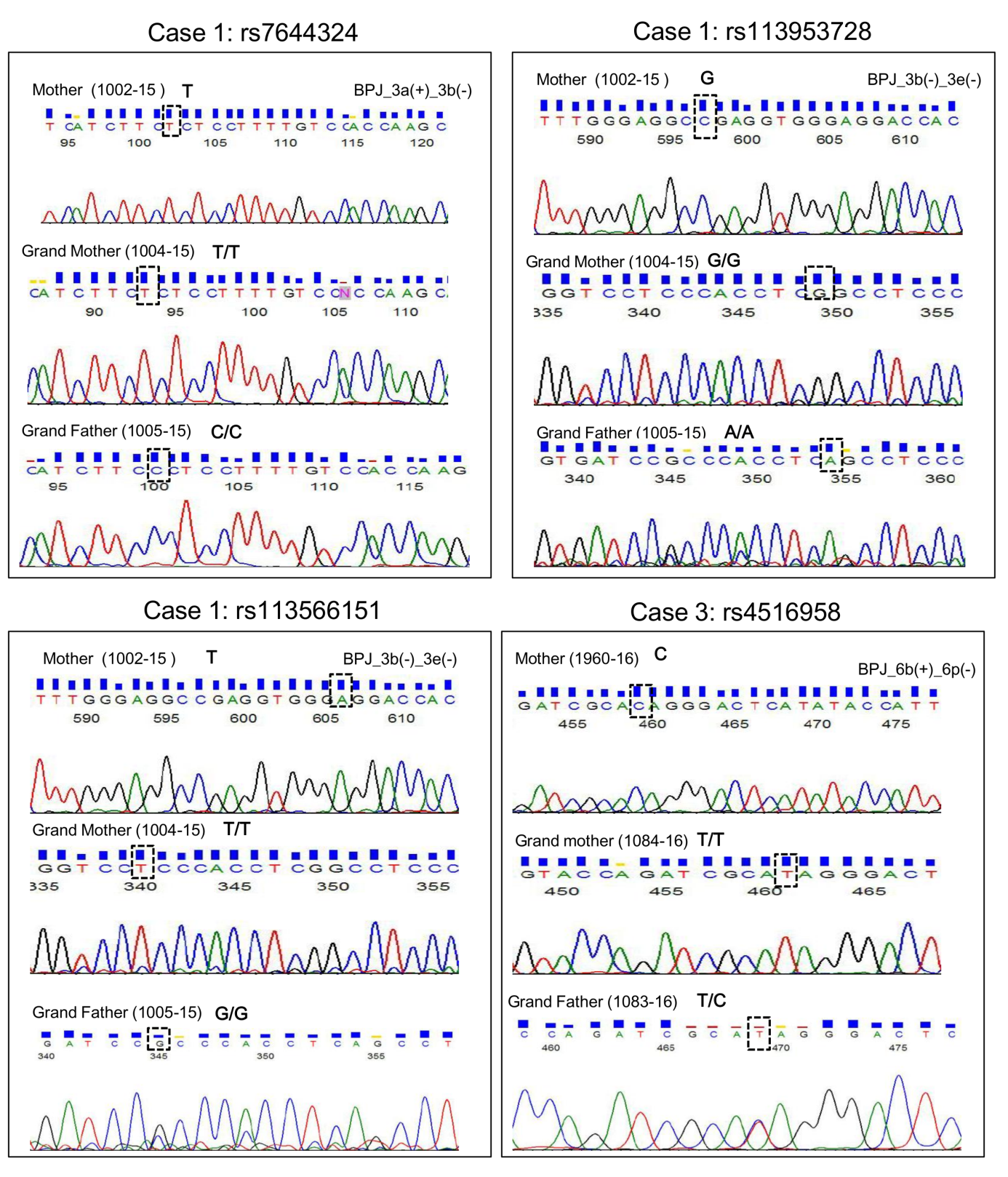
**

Case 1 and 3: DNA of maternal grandparents was screened for the SNPs detected in the case-specific Sanger sequencing data. In case 1, three informative SNPs, rs7644324, rs113953728 and rs113566151 (indicated by dashed rectangles) showed the maternal origin of the chromothripsed chromosome 3 of the mother (1002-15). In case 3, a single informative SNP, rs4516958, revealed a paternal origin of the derivative chromosome 6 of the mother (1960-16).

**Supplementary Figure S5**

**
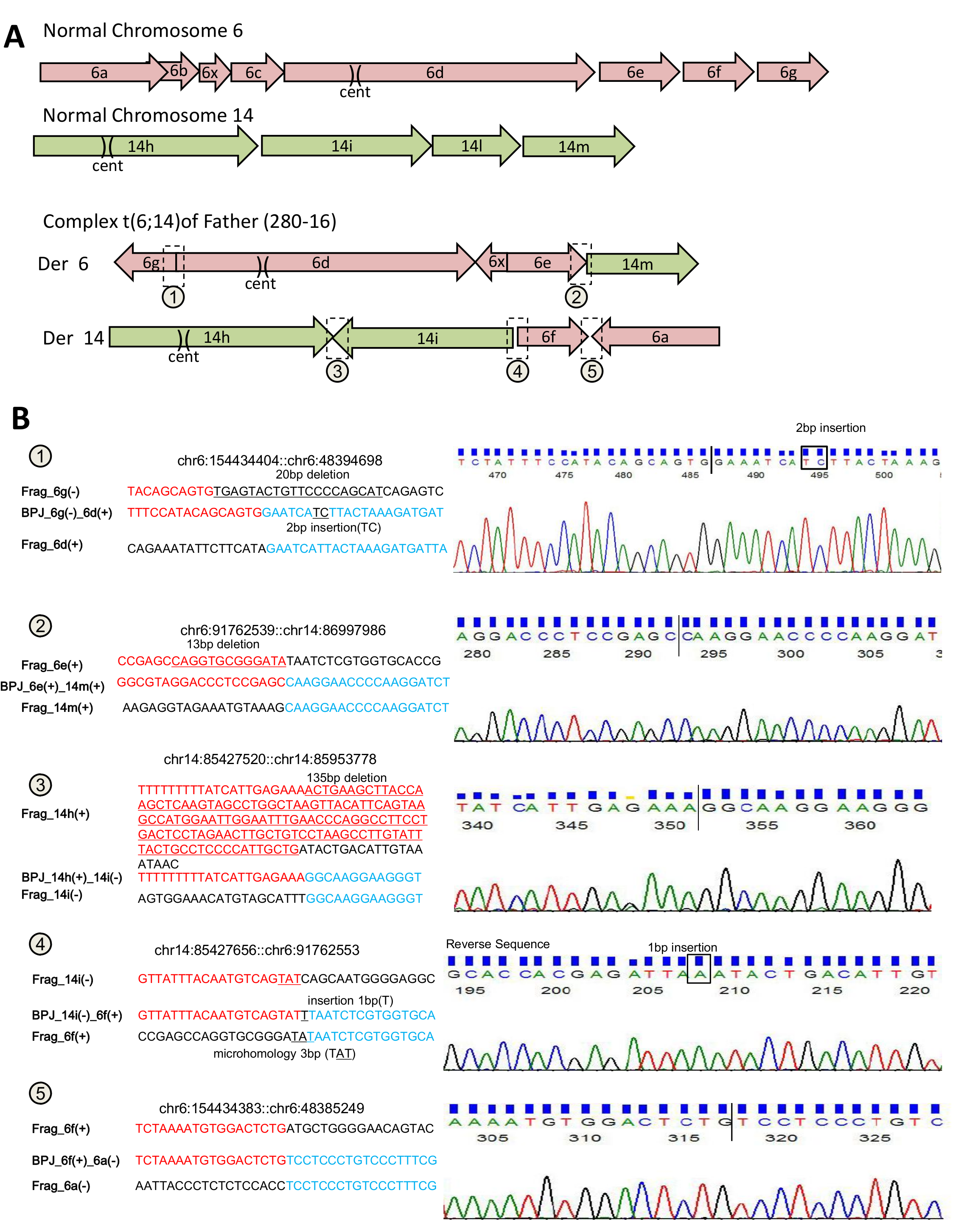
**

Case 2. A: chromosomal rearrangement of t(6;14) in father (280-16) is schematically illustrated. Inverted fragments, 6g, 6x, 6a and 14i are indicated with left arrowheads. Fusion junctions that have been validated by Sanger are numbered from 1 to 5 and indicated by dashed rectangles.

B: the reference sequences of each shattered fragment (red and blue) are shown. Inverted fragments are indicated with minus (-) sign. Deleted and inserted sequences and the only microhomology at the breakpoint junctions are underlined in the text. In total we detected three small deletions (numbers in circles) at the following fusion junctions: BPJ_6g(-)_6d(+) (20bps, junction 1), BPJ_6e(+)_14m(+) (13bps, junction 2), BPJ_14h(+)_14i(+) (135bps, junction 3), two small insertions at BPJ_6g(-)_6d(+) (2bps, junction 1) and BPJ_14i(-)_6f(+) (1bp, junction 4), one microhomology at BPJ_14(i)_6f(+) (3bps, junction 4); and one blunt fusion at BPJ_6f(+)_6a(-) (junction 5). Cent: centromere, Der: derivative

**Supplementary Figure S6**

**
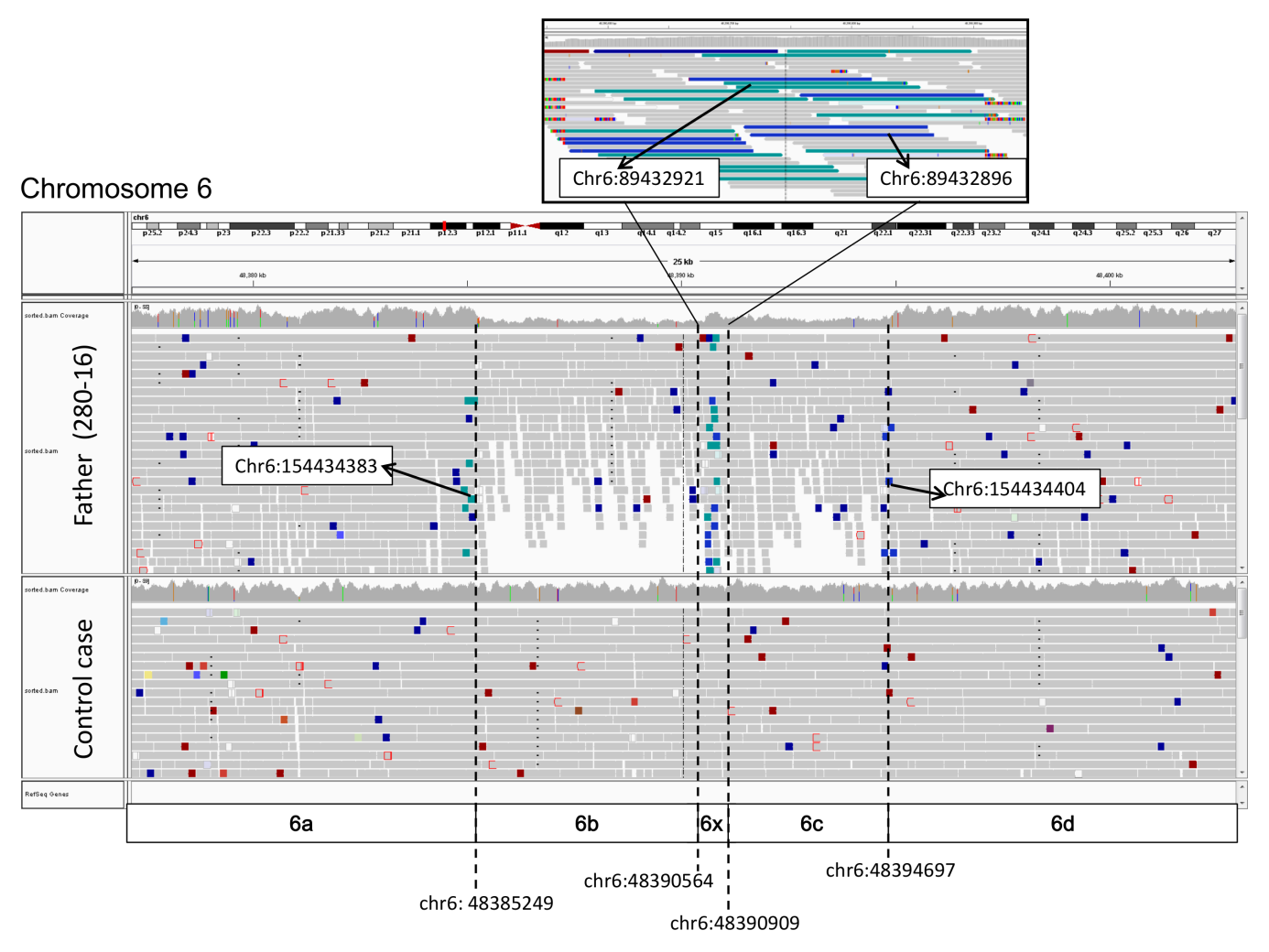
**

Case 2. IGV visualization of a window of genomic region chr6:48,377,156-48,402,966 of father (280-16, case 2) involving shattered fragments; 6a, 6b, 6x, 6c and 6d (indicated in rectangles); a control case is shown below. The breakpoints of each fragment are indicated with dashed lines. Discordant reads (green and dark blue reads) at the fragment 6x (chr6:48390564-48390909) were highlighted in the box. The green discordant read at chr6:48390564 mapped to Chr6:89432921, while the blue discordant read at chr6:48390909 mapped to chr6:89432896 indicating inverted translocation of the fragment 6x, originally at 6p12.3, into the 6q15. Deletion of fragments 6b and 6c was demonstrated by their decreased coverage (21,2x) in comparison with the average coverage of whole chromosome 6 (42,1x) of father (280-16) and same region of the control case (43x). The discordant read (green) at chr6:48385249 (right side of fragment 6a) mapped to Chr6:154434383. The discordant read (dark blue) at chr6:48394697 (left side of fragment 6d) was mapped to Chr6:154434404.

**Supplementary Figure S7**

**
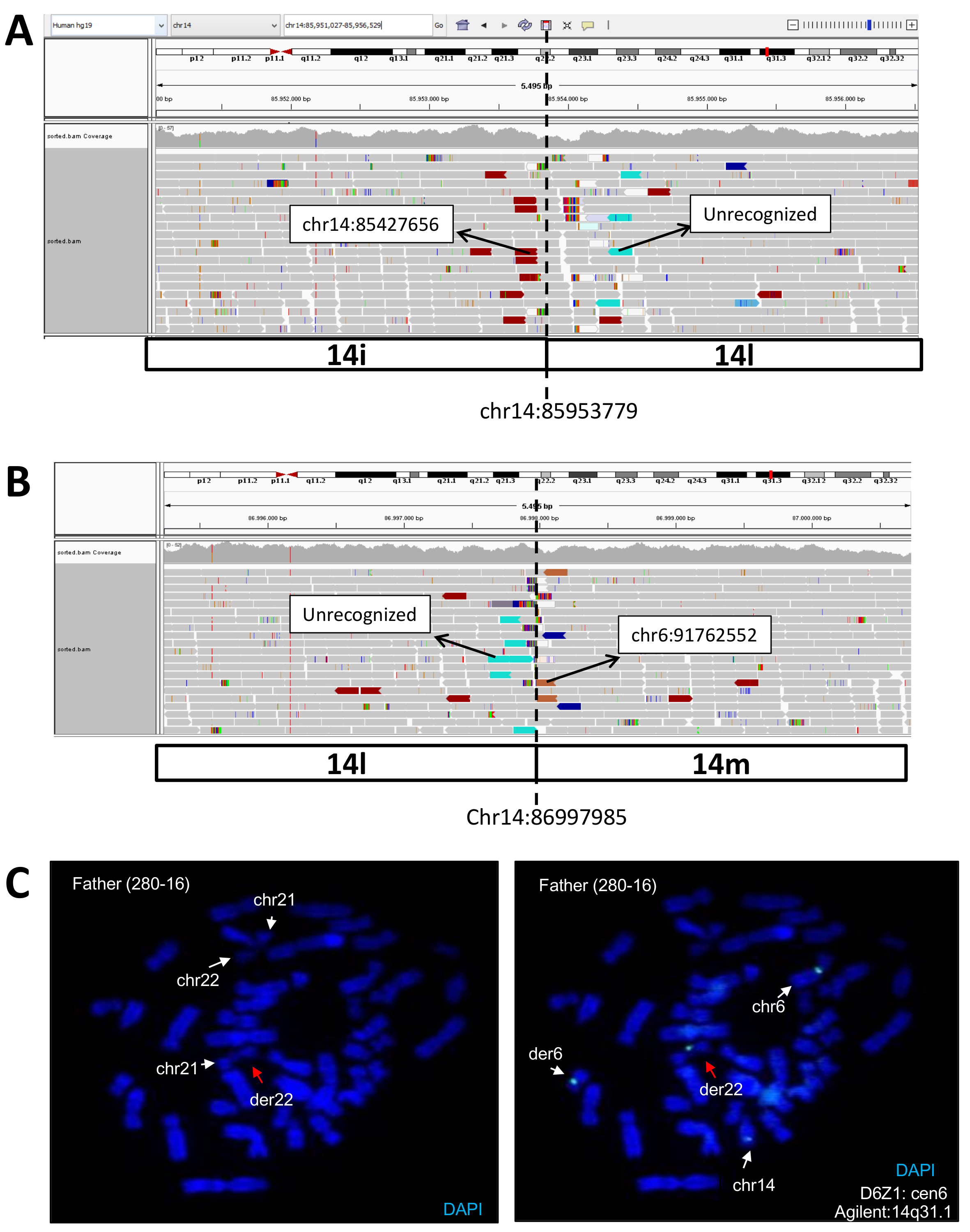
**

Case 2. A, B: IGV visualization of fragments 14i, 14l and 14m of chromosome 14 of the father (280-16). Dashed lines indicate breakpoints within fragments. A: the breakpoint between fragments 14i and the left side of fragment 14l is located at chr14:85953779. The breakpoint of fragment 14i is defined by the discordant reads (dark green) which mapped to chr14:85,427,656. The breakpoint belonging to the left side of fragment 14l was defined by discordant reads (turquoise) that mapped to an unrecognized region of the genome. B: the window of genomic region of chr14:86,996,973-86,998,996 including fragments 14l and 14m is shown. The breakpoint between the right site of fragment 14l and the left side of the fragment 14m was located at chr14:86997985. The discordant reads (orange) belonging to fragment 14m, was mapped to chr6:91762552, while the discordant reads (turquoise) belonging to the fragment 14l, mapped to an unrecognized portion of the genome. C: FISH was performed with custom Agilent probe (14q31.1, green) targeting fragment 14l and D6Z1 (cen6, green) (Cytocell). The metaphase is stained with DAPI. In the left, chromosomes 21 and 22 are indicated. Red arrow illustrates chromosome 22 where fragment 14I was located.

**Supplementary Figure S8**

**
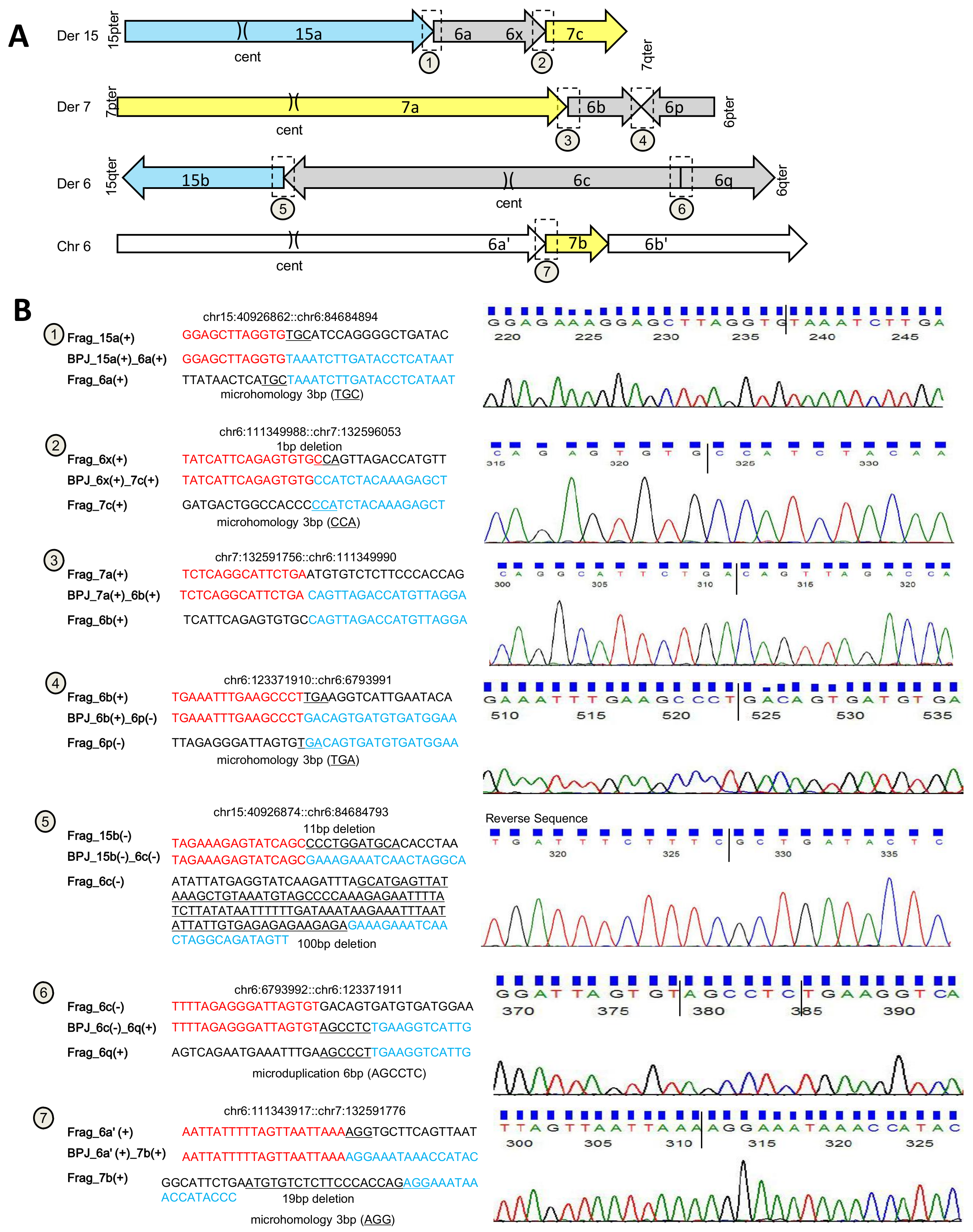
**

Case 3. Rearrangements of t(6;7;15) in the mother (1960-16). A: inverted fragments, 6c, 6p and 6q, are indicated with left arrowheads. Fusion junctions, indicated by dashed rectangles, are numbered within circles from 1 to 7. B: Sanger validation of each breakpoint junctions of the shattered fragments and their reference sequences prior to the chromothripsis (red and blue) are shown. The inverted fragments are indicated with minus (-) sign. Microduplication and microhomology in the breakpoint junctions are underlined in the text. In total we observed four microhomologies at the following fusion junctions: BPJ_15a(+)_6a(+) (3bps, junction 1), BPJ_6x(+)_7c(+) (3bps, junction 2), BPJ_6b(+)_6p(-) (3bps, junction 4), BPJ_6a’(+)_7b(+) (3bps, junction 7), three microdeletions at BPJ_6x(+)_7c(+) (1bp, junction 2), BPJ_15b(-)_6c(-) (11bps and 100bps, junction 5) and BPJ_6a’(+)_7b(+) (19bps, junction 7), one microduplication BPJ_6c(-)_6q(+) (6bps, junction 6). At the breakpoint junction BPJ_6c(-)_6q(+), the microduplicated fragment (AGCCTC) was different compared to its reference sequence (AGCCCT) for 2bps at the 3' end, possibly indicating a replication error while handling repair.

**Supplementary Figure S9**

**
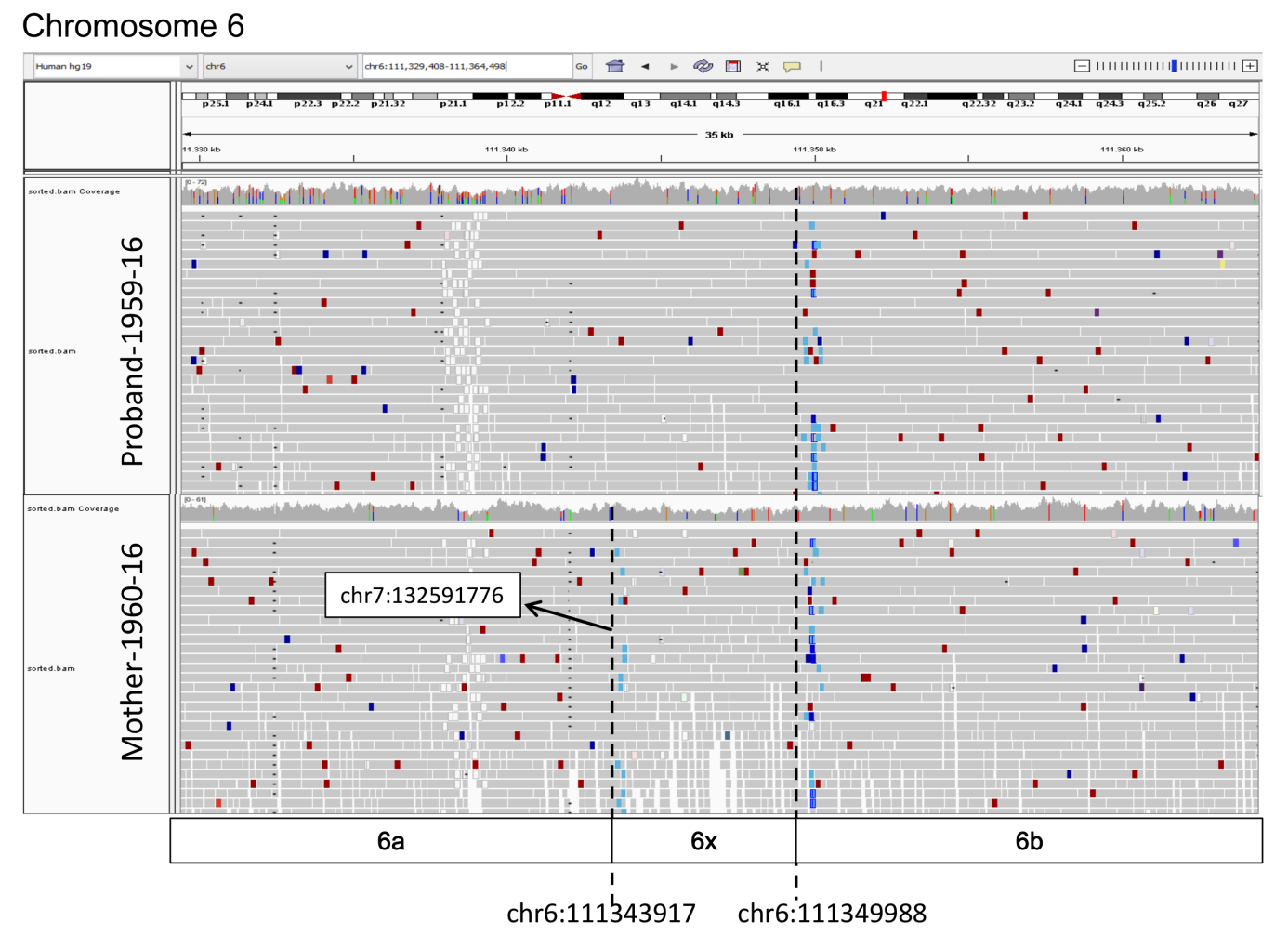
**

Case 3. IGV view of breakpoints of chromosome 6 of mother (1960-16, case 3) and proband (1959-16) encompassing the chromothriptic fragments 6a, 6x and 6b (indicated in rectangles). Breakpoints are indicated with dashed lines and discordant reads are highlighted (blue). The breakpoint of 6a-6x located at chr6:111343917, mapping to chr7:132591776 was present in the mother and absent in the proband.

**Supplementary Figure S10**

**
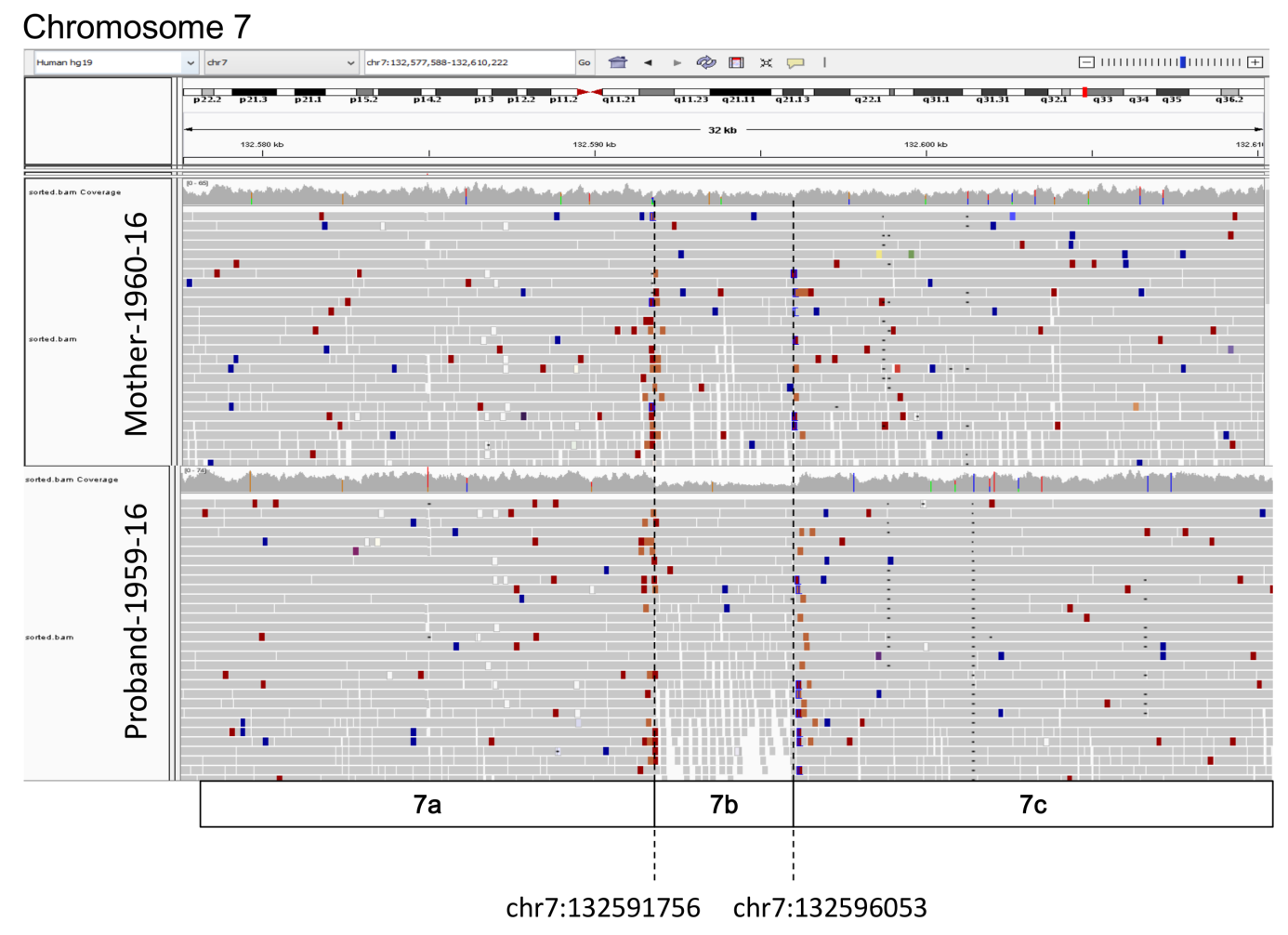
**

Case 3. IGV view of breakpoints of chromosome 7 of mother (1960-16) and proband (1959-16) producing fragments 7a, 7b and 7c (indicated in rectangles). Breakpoints are highlighted with dashed lines. Deletion of fragment 7b in the proband is shown by decreased coverage (16,3x), as detected by CNVnator. The same region was in balanced state in the mother (30x).

**Supplementary Figure S11**

^~~
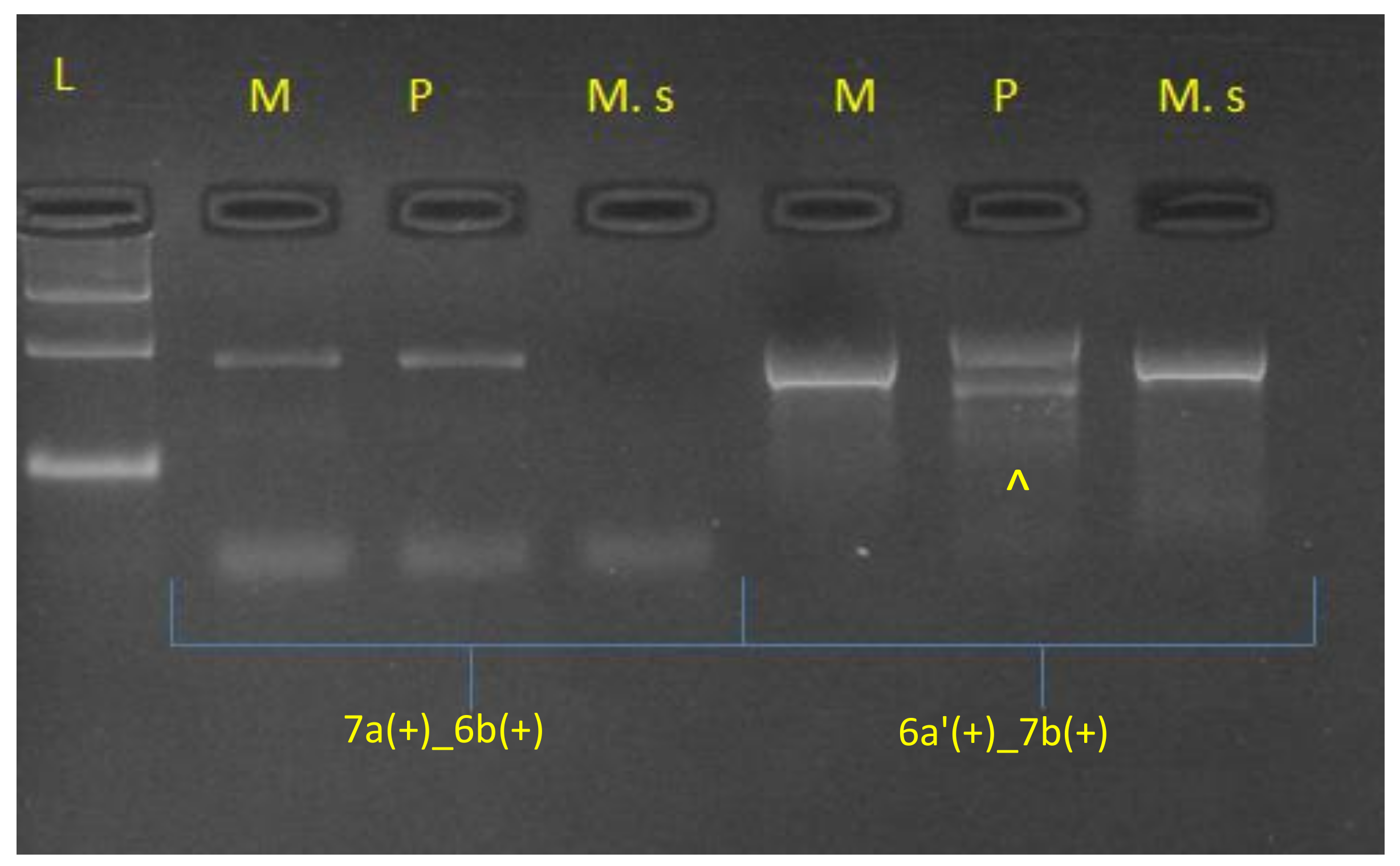
~~^

Case 3. Breakpoint specific PCR for the fusion junctions 7a(+)_6b(+) and 6a'(+)_7b(+) on proband (1959-16) and mother (1960-16). P: proband, M: Mother (peripheral blood DNA), M.s: Mother (saliva derived DNA), L: Ladder (GelPlot 100bp plus ladder-Qiagen), (^): signifies non-specific amplification. The fusion junction 7a(+)_6b(+) was detected both in mother (peripheral blood DNA) and proband, while it was absent in M.s. Mother specific fusion junction 6a'(+)_7b(+) was detected in both DNA samples of the mother, while it was absent in the proband.

**Supplementary Appendix S1**

**Phenotype Genotype Correlation**

**Case 1**

The neurodevelopmental phenotype of the proband (1001-15) may be the consequence of different genomic alterations: (1) dosage effects of the genes involved in the 5Mb deletion and 10 M duplication, (2) disruption of TADs either as a consequence of the CNVs or the inherited rearrangement.

(1) The 5 Mb deletion involved 73 genes (Table S4). Among these, 6 of them, *SLC7A14, SLC2A2, TNIK, PLD1, GHSR* and *SPATA16* were linked with a known OMIM disease. However, none of them matched with proband's phenotype. According to haploinsufficiency scores (Table S4), %HI <10% and pLI >0.9, 9 out of 24 genes, *SEC62, PHC3, TNIK, FNDC3B, CLDN11, EIF5A2, ECT2, NLGN1, NAALADL2,* exhibit haploinsufficiency. In DECIPHER, 8 patients, all having smaller deletions within the 5Mb deleted region, were reported for developmental defects together with varied clinical features. DECIPHER patient, 257342, having a *de novo* smaller deletion (1,08Mb) involving *ECT2, NLGN1, SPATA16* showed several developmental abnormalities in common to our patient as intellectual disability, delayed speech and behavioural abnormalities. In three other cases with developmental delay, 287264, 270579 and 283752, the overlapping deleted region encompassed a single gene, *NLGN1*. However in these cases the deletion was inherited from one of the parent which was either unaffected or with an unknown phenotype, thus making doubtful a causative role for *NLGN1* haploinsufficiency. The 10Mb duplicated region encompassed 181 genes (Table S4). We checked ClinGen for dosage sensitivity, however triplosensitivity scores for the duplicated genes were not available. In DECIPHER, the only patient (253876) having a comparable size duplication (7,63Mb) was with intellectual disability, short stature and abnormalities of neck and limbs. In conclusion we could not find any obvious association between the patients clinical features and haploinsufficiency or duplication of a specific gene.

(2) Four breakpoints shared by both mother and affected son were predicted to disrupt TADs, likely affecting the genes PLXNA1, SEC62, BFSP2 and TERC in lymphoblastoid cells (Table S3). Among these genes, TERC was a strong candidate disease-gene because it was expressed in whole blood and was associated with known autosomal dominant diseases; dyskeratosis congenita (MIM#127550) and aplastic anemia (MIM#614743). However, both mother and proband were negative for the predicted TAD-phenotype correlation, possibly due to incomplete penetrance or more complex factors involved in the disease manifestation. In the mother, the breakpoint chr8:32716994, which located at an active transcription site in post mortem brain (as suggested by H3K4Me3), was predicted to disrupt TAD in neural precursor cells, likely affecting *NRG1* which is expressed in neural cells. *NRG1* is not associated with a known disease, however it is reported with high haploinsufficiency score.

**Case 2**

Both father (280-16) and new-born proband (256-16) were healthy individuals according to current clinical investigations. In father, the disrupted genes *RNGTT* and *OPRM1*, both having high haploinsufficiency scores, were not associated with a known disease. Five breakpoints were predicted to disrupt two TADs, likely affecting disease-associated genes *RHAG* and *MAP3K7* in neural cell. However, *RHAG* is mainly expressed in whole blood and it belongs to a TAD that is not disrupted in lymphoblastoid (GM12878) cells, suggesting its disruption non-pathogenic. While *MAP3K7*, which was expressed in neural cells, was associated with cardiospondylocarpofacial syndrome (MIM#157800) and frontometaphyseal dysplasia 2 (MIM#617137) and had a high haploinsufficiency score, suggesting its misregulation likely pathogenic. On the other hand, bona fide absence of any disease phenotype in the father, disagrees with the pathogenesis of the predicted TAD disruption.

**Case 3**

In this case, the 14-year-old proband (1959-16) was referred because of psychomotor delay, while the mother (1960-16) was healthy but with a low-level job compared to the socio-economic position of the family of origin. None of the disrupted genes were associated with a known autosomal dominant disease. Regarding to the chromothripsis as characterized by WGS, the only genotypic difference between mother and son concerns the 4,2kb region belonging to fragment 7b that is deleted in the proband, while it was insertionally translocated in the mother. This region involves a part of intron 4 of *CHCHD3*, a gene encoding a protein located at the inner membrane of mitochondria. Silencing of *CHCHD3* showed its essential role in mitochondrial morphology and functioning (Darshi et al., 2011). Both in mother and son, translocation/deletion of fragment 7b is expected to result in the same biological impact in the gene functioning if there is any. Thereafter we may assume that the condition of mosaicism of the complex rearrangement in the mother as indirectly demonstrated by breakpoint cloning in two different DNA materials, is responsible for the psychomotor delay evident in the child. In order to find a further genotype-phenotype association and mainly to explain the resulting phenotype of the proband, we checked each breakpoints for TAD disruption and potential candidate disease genes within the disrupted TADs. One of the inherited breakpoint; chr15:40926862, disrupts a TAD which might affect *TTBK2* in neural precursor cells. *TTBK2*, having a high haploinsufficency score, is associated with spinocerebellar ataxia (MIM#604432). Autosomal dominant spinocerebellar ataxias are a heterogeneous group of neurodegenerative disorders characterized by several clinical signs including poor coordination, abnormal eye movements, impairment of speech and swallowing, and pyramidal signs (Houlden et al., 2007). Although the onset of the syndrome is in third or fourth decades, the 42–year-old mother is so far totally healthy although sharing the same breakpoint. Another inherited breakpoint at chromosome 6, chr6:84684793, disrupted a TAD in neural cells likely affecting *TBX18*. *TBX18* is associated with congenital anomalies of kidney and urinary tract (MIM#143400). We checked Hi-C data of bladder (Zhang et al., 2017), which is the most relevant organ for the disease of interest among the provided Hi-C data of different cell sources. TAD disruption for the same breakpoint involved *TBX18* also in bladder. As a result, TAD disruption as a cause of breakpoint at chr6:84684793 might be potential candidate for the pathogenesis of hypospadias detected in proband.

References

Darshi, M., Mendiola, V. L., Mackey, M. R., Murphy, A. N., Koller, A., Perkins, G. A., … Taylor, S. S. (2011). ChChd3, an inner mitochondrial membrane protein, is essential for maintaining Crista integrity and mitochondrial function. *Journal of Biological Chemistry*, *286*(4), 2918–2932. http://doi.org/10.1074/jbc.M110.171975

Houlden, H., Johnson, J., Gardner-Thorpe, C., Lashley, T., Hernandez, D., Worth, P., … Wood, N. W. (2007). Mutations in TTBK2, encoding a kinase implicated in tau phosphorylation, segregate with spinocerebellar ataxia type 11. *Nature Genetics*, *39*(12), 1434–1436. http://doi.org/10.1038/ng.2007.43

Zhang, Y., An, L., Hu, M., Tang, J., Yue, F. (2017). HiCPlus: Resolution Enhancement of Hi-C interaction heatmap. *bioRxiv*, 112631. http://doi.org/10.1101/112631
